# Reorganization of Lipid Diffusion by Myelin Basic Protein as Revealed by STED Nanoscopy

**DOI:** 10.1016/j.bpj.2016.04.047

**Published:** 2016-06-07

**Authors:** Olena Steshenko, Débora M. Andrade, Alf Honigmann, Veronika Mueller, Falk Schneider, Erdinc Sezgin, Stefan W. Hell, Mikael Simons, Christian Eggeling

**Affiliations:** 1 Cellular Neuroscience, Max Planck Institute of Experimental Medicine, Göttingen, Germany; 2 Department of Nanobiophotonics, Max Planck Institute for Biophysical Chemistry, Göttingen, Germany; 3 Centre for Neural Circuits and Behaviour, University of Oxford, Oxford, United Kingdom; 4 Max Planck Institute of Molecular Cell Biology and Genetics, Dresden, Germany; 5 MRC Human Immunology Unit, Weatherall Institute of Molecular Medicine, University of Oxford, Oxford, United Kingdom; 6 Institute of Neuronal Cell Biology, Technical University Munich, Munich, Germany

## Abstract

Myelin is a multilayered membrane that ensheathes axonal fibers in the vertebrate nervous system, allowing fast propagation of nerve action potentials. It contains densely packed lipids, lacks an actin-based cytocortex, and requires myelin basic protein (MBP) as its major structural component. This protein is the basic constituent of the proteinaceous meshwork that is localized between adjacent cytoplasmic membranes of the myelin sheath. Yet, it is not clear how MBP influences the organization and dynamics of the lipid constituents of myelin. Here, we used optical stimulated emission depletion super-resolution microscopy in combination with fluorescence correlation spectroscopy to assess the characteristics of diffusion of different fluorescent lipid analogs in myelin membrane sheets of cultured oligodendrocytes and in micrometer-sized domains that were induced by MBP in live epithelial PtK2 cells. Lipid diffusion was significantly faster and less anomalous both in oligodendrocytes and inside the MBP-rich domains of PtK2 cells compared with undisturbed live PtK2 cells. Our data show that MBP reorganizes lipid diffusion, possibly by preventing the buildup of an actin-based cytocortex and by preventing most membrane proteins from entering the myelin sheath region. Yet, in contrast to myelin sheets in oligodendrocytes, the MBP-induced domains in epithelial PtK2 cells demonstrate no change in lipid order, indicating that segregation of long-chain lipids into myelin sheets is a process specific to oligodendrocytes.

## Introduction

Myelin is a highly specialized membrane that forms a continuous, multilayered stack of tightly packed membrane, ensheathing the axons in the central and peripheral nervous systems (1, 2). In contrast to most plasma membranes, the myelin membrane contains a high proportion of lipids, which account for 80% of its dry weight (3, 4). Remarkably, the myelin membrane is enriched in cholesterol, plasmalogens, and galactosylceramide lipid species, with a high fraction of lipids with long-chain fatty acids (5, 6, 7). The self-segregation of these very-long-chain glycosphingolipids from other, shorter lipids may be a driving factor in the generation of the highly ordered myelin membrane (8). This high content of tightly packed lipids is essential for the electrical insulating properties of the myelin membrane that are required to increase the propagation speed of the impulses along myelinated fibers (1, 9).

The key molecule that organizes the structure of myelin membrane sheets is myelin basic protein (MBP) (10, 11). This intrinsically disordered polypeptide chain has a strong basic character and polymerizes into a cohesive mesh-like protein network when bound to the inner leaflet of membrane bilayers (12, 13, 14). Within the cytoplasmic space of the compact myelin sheath, MBP forms a size-selective barrier that prevents diffusion of most cytosolic and membrane proteins into the compact myelin sheath region (4, 15). In addition to its specific lipid composition and low amount of proteins, the compact myelin sheath also lacks an actin-based cytocortex (4, 16, 17).

Due to these characteristics, as well as its abundance and ordered laminar structure, myelin has served as a model membrane for many biochemists and biophysicists (18, 19). However, it is not yet clear how the large amount of structural information on myelin relates to the general principles of membrane dynamics and thus membrane bioactivity.

Over the past few years, investigators have refined the basic model of membrane structure organized as homogeneous fluid mosaics (20) by including the concept of heterogeneity. Lipid-lipid and lipid-protein interactions, protein oligomerization, and the underlying cortical actin cytoskeleton are major driving forces that compartmentalize the plasma membrane (21, 22, 23). Prominent examples include lipid microdomains, caveolae, coated pits, and actin cytoskeleton-induced subcompartments (24, 25, 26, 27, 28). Due to this patchwork organization of the plasma membrane, diffusion of molecules is in many cases not free, but anomalous (21, 24, 29, 30, 31, 32). For example, interactions with less mobile entities such as proteins lead to transient slowdowns in diffusion (trapping) (22, 29, 31, 33), whereas actin-induced compartments result in temporary confinements within 30–300 nm compartments (hop diffusion) (24, 30, 34, 35). All of these structural and diffusional heterogeneities rule the bioactivity of membrane molecules (36).

Recent studies have investigated plasma membrane dynamics by exploiting the nanoscale spatial resolution provided by optical stimulated emission depletion (STED) microscopy (or nanoscopy) (37) in conjunction with fluorescence correlation spectroscopy (STED-FCS) (29, 38) to observe anomalous diffusion of membrane molecules, such as the transient binding of lipids to other membrane constituents (31, 32, 39, 40). STED-FCS enabled investigators to make a direct distinction between free and anomalous diffusion (29). These studies demonstrated that the diffusion of phosphoglycerolipids without hydroxyl-containing headgroups was relatively free, showing only weak interactions within the membrane. The strongest confinements among the tested lipids were observed for sphingolipid analogs that showed cholesterol-assisted and cytoskeleton-dependent transient trapping, most probably due to transient binding to immobile or slow-moving proteins (29, 31, 32).

Taking into account the peculiar organization of the myelin membrane, in this work we used STED-FCS in combination with fluorescent lipid analogs to address the question of how MBP organizes heterogeneity in lipid diffusion. We observed significantly faster and less anomalous lipid diffusion both in oligodendrocytes (of which MBP is a ubiquitous component) and in MBP-rich domains of MBP-transfected epithelial PtK2 cells. Using two different polarity-sensitive fluorescent lipid reporters, we further tested whether these MBP-rich domains are characterized by a higher molecular lipid order, as was previously reported for oligodendrocytes. However, our data showed no difference in order between inside and outside of the domains.

## Materials and Methods

Preparation of oligodendrocytes and epithelial PtK2 cells on glass coverslips, incorporation of the fluorescent lipid analogs into the plasma membrane of the living cells via bovine serum albumin complexes, the PtK2-MBP assay, immunolabeling and confocal imaging details, STED-FCS measurement conditions and procedures, the STED-FCS setup and its calibration, the spectral imaging setup, and the FCS analysis are described in the Supporting Materials and Methods.

## Results

**Figure 1:**
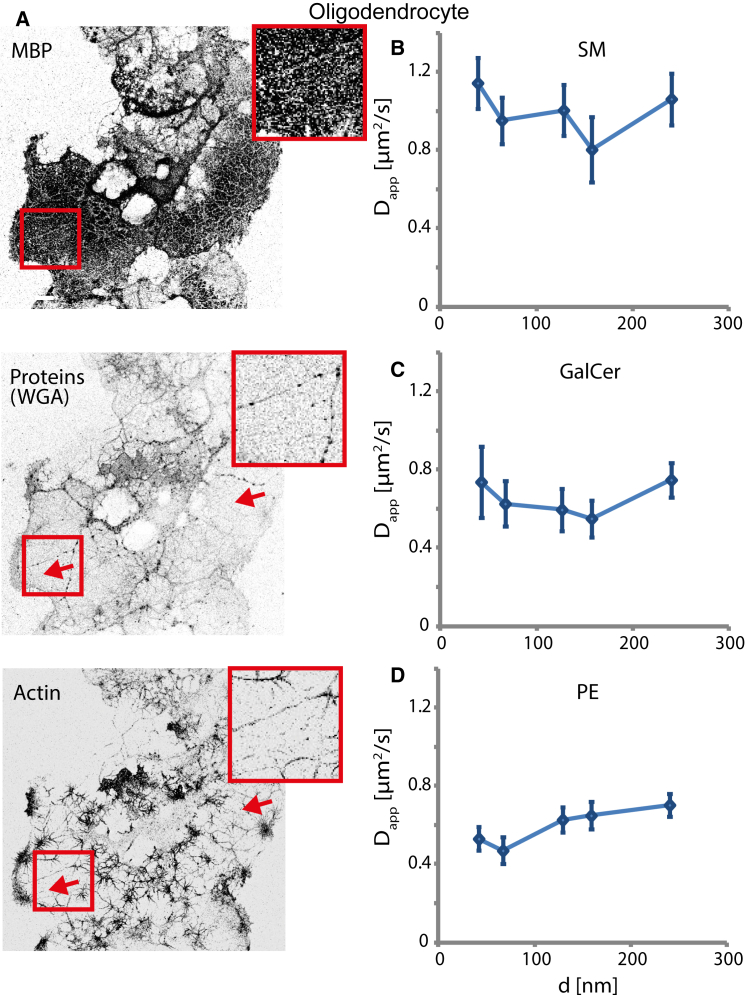
Lipids diffuse approximately freely in oligodendrocyte membrane sheets. (*A*) Confocal fluorescence images (50 × 50 *μ*m^2^, inverted grayscale from *white* (low signal) to *black* (high signal), contrast adapted between the different images to highlight differences) of cultured oligodendrocytes fixed with 4% paraformaldehyde and 0.25% glutaraldehyde on day 5. The images show cell-surface fluorescence staining for MBP (*top*), sialic acid and N-acetylglucosaminyl residues with WGA-Alexa488 conjugate for general protein staining (*middle*), and after permeabilization and fluorescence immunostaining for filamentous actin with rhodamine-phalloidin (*bottom*), indicating that proteins and actin were depleted from the membrane sheet areas (representatively indicated by the *red arrows* and *red flanked insets*, which show zoom-ins of the areas marked with a *red box*). (*B–D*) STED-FCS analysis, showing the dependence of D_app_ on the diameter *d* of the microscope’s observation spot for the fluorescent lipid analogs SM (*top*), GalCer (*middle*), and PE (*bottom*) in the myelin sheets at 37°C. The roughly constant values indicate approximately free diffusion. The plots show mean ± standard error of the mean (SEM) (*n* = 15 cells). To see this figure in color, go online.

To study lipid diffusion in myelin, we first used primary cultures of oligodendrocytes as an experimental system. When cultured on glass coverslips, oligodendrocytes form large myelin membrane sheets (Fig. 1). This system satisfies many of the essential requirements for studying the structure of the myelin membrane. First, the membrane sheets resemble in vivo compact myelin in their lipid and protein compositions. The main components of compact myelin localize to membrane sheets, such as the MBP, while most other proteins are excluded, as shown by staining for sialic acid and N-acetylglucosaminyl protein residues (wheat germ agglutinin (WGA); Fig. 1 *A*). In addition, as shown previously (16, 17), staining for actin revealed no distinct actin cytoskeleton network within the sheets (Fig. 1 *A*). As the cortical actin network is one of the main modulators of molecular diffusion in the plasma membrane, we measured lipid mobility in the membrane sheets. For this purpose, we incorporated different lipid analogs labeled with the lipophilic organic dye Atto647N into the plasma membrane of living oligodendrocytes by incubation with lipid-bovine serum albumin complexes, and monitored their diffusion pattern using STED-FCS. All measurements reported here were performed in living cells at 37°C. Other than the unexpected finding in previous studies that Atto647N-labeled saturated lipid analogs partitioned into the liquid-disordered (instead of the liquid-ordered) phase in model membranes (31, 41), this label has not been found to have any notable effect on the interactions of lipids in live cells, at least as observed by STED-FCS (29, 31, 32). We chose to use fluorescent lipid analogs of saturated phosphatidylethanolamine (referred to as phosphoethanolamine or simply PE, acyl chain length C15, label at headgroup) and saturated sphingomyelin (SM, C12, labeling via acyl-chain replacement) for our diffusion studies because their behavior in epithelial cells and fibroblasts has been shown to be significantly distinguishable (29, 31, 32). In addition, we chose a fluorescent analog of a highly abundant myelin lipid, galactosylceramide (GalCer, C13, labeling via acyl-chain replacement).

STED-FCS allows one to determine the apparent diffusion coefficient, D_app_, for different sizes, i.e., different diameters, *d*, of the observation spot, typically *d* = 40–240 nm (31). Although D_app_ values determined from FCS data of the (diffraction-limited) confocal observation spot (*d* = 240 nm) only report on the average (relatively long-scale) mobility of the lipids, the observation and comparison of D_app_ values for different sizes of the observation spot highlight characteristics of the diffusion pattern (29, 31, 42). The independence of the observed D_app_ with respect to the size of the observation spot is indicative of free diffusion (31). In this case, diffusion is not hindered (at least on the spatial scales investigated, i.e., *d* > 40 nm) and is only limited by the characteristic viscosity of the medium in which the molecules diffuse. Conversely, when D_app_ is observed to increase as the size of the observation spot is increased, the molecules experience trapping, i.e., diffusion is transiently slowed down or the molecules are arrested on small spatial scales due to, for example, transient interactions with slower-moving or immobilized binding partners (31, 42). For small observation spots, the transit time of trapped molecules is much more dominated by the trapping time and thus leads to a decrease of the measured D_app_ (in contrast to the real instant diffusion coefficient, which stays the same in between the traps). This should not be confused with findings from single-particle tracking (SPT) experiments, where trapping usually results in reduced long-scale (or macroscopic) diffusion coefficients compared with the large (fast) real instant or microscopic diffusion coefficient in between traps as measured on short spatial scales (or for small time steps). These two techniques (STED-FCS and SPT) indeed assess confined diffusion differently, as highlighted by the fact that hopping diffusion and trapping diffusion are indistinguishable from the perspective of SPT, whereas in STED-FCS these two modalities of anomalous diffusion are observed fundamentally differently (32, 43).

Our STED-FCS recordings on oligodendrocytes revealed that all lipid analogs investigated here diffused approximately freely in membrane sheets, with D_app_ ≈ 0.70–1.0 *μ*m^2^/s, the same range as the average mobility that was previously reported in conventional confocal FCS recordings of a Bodipy-labeled lipid analog (8) (Fig. 1, *B–D*). Although previous STED-FCS studies showed that PE diffused approximately freely in the plasma membrane of epithelial cells and fibroblasts, conversely, they also showed that SM and GalCer underwent trapping diffusion in those cells (29, 31, 32).

**Figure 2:**
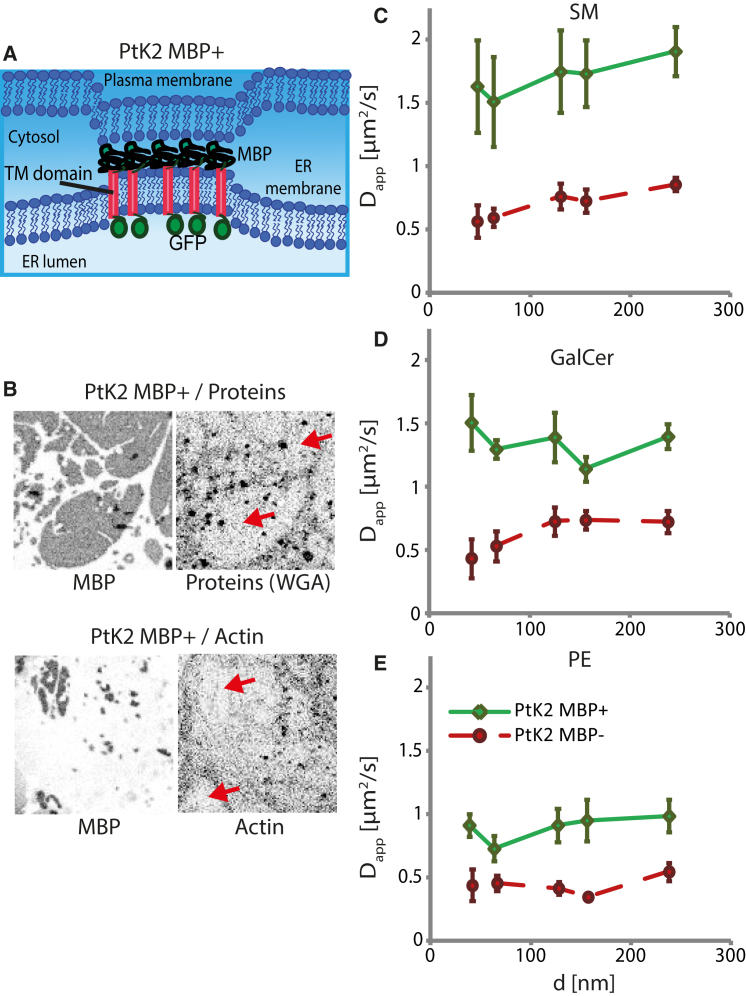
The formation of MBP-mediated membrane patches alters lipid diffusion in PtK2 cells. (*A*) Schematic drawing of MBP-mediated membrane domains: upon expression in PtK2 cells, the MBP-expressing chimeric fluorescent protein GFP-TM-MBP forms ER-plasma membrane patches that are depleted of glycoproteins and cortical actin cytoskeleton. (*B*) Confocal fluorescence images (6 × 6 *μ*m^2^, inverted grayscale from *white* (low signal) to *black* (high signal), contrast adapted between the different images to highlight differences) of cultured and GFP-TM-MBP transfected epithelial PtK2 cells (PtK2 MBP+) fixed with 4% paraformaldehyde and 0.25% glutaraldehyde, at 16 h after transfection. The panel shows fluorescence immunostaining for sialic acid and N-acetylglucosaminyl protein residues with WGA-Alexa488 (panel labeled with proteins (WGA)) and filamentous actin with rhodamine-phalloidin label (panel labeled with actin), and the respective GFP-TM-MBP images (panels labeled with MBP). As for membrane sheets, proteins and F-actin were depleted from the MBP-mediated membrane patches (representatively indicated by the *red arrows*). (*C–E*) STED-FCS analysis, showing the dependence of D_app_ on the diameter *d* of the microscope’s observation spot for the fluorescent lipid analogs SM (*C*), GalCer (*D*), and PE (*E*) in PtK2 MBP+ (*green*) and PtK2 MBP− (*red dotted*) cells. Bars show SEM (*n* = 15 cells). All measurements were performed at 37°C. To see this figure in color, go online.

These findings pinpoint the peculiar nature of myelin membrane sheets. We previously showed that MBP expression as a chimeric construct (green fluorescent protein (GFP) labeled (GFP-TM-MBP)) generates connections between the membrane of the endoplasmic reticulum (ER) and the plasma membrane in epithelial PtK2 cells (Fig. 2 *A*) (13). These micrometer-sized, protein-poor domains resemble compact myelin in terms of protein composition (enriched in MBP and deprived in N-acetylglucosaminyl protein residues) and also lack a cortical actin cytoskeleton (Fig. 2 *B*). To investigate the specific role of MBP in modulating lipid diffusion in membranes, we took advantage of this system of GFP-TM-MBP-transfected PtK2 cells and observed diffusion via STED-FCS inside and outside of MBP domains (Fig. 2, *C–E*). We found that all three lipid analogs (SM, GalCer, and PE) diffused at least twice as fast within the domains (PtK2 MBP+) as compared with the control (nonexpressing) PtK2 cells (PtK2 MBP−). Moreover, diffusion of all lipid analogs appeared to be free within MBP domains, which is in contrast to the diffusion of SM and GalCer in the control PtK2 MBP− cells.

We have to note that we cannot distinguish between lipid diffusion in the outer and inner leaflets of the plasma membrane, or, in the case of the PtK2 MBP+ domains, between diffusion of the lipid analogs in the plasma membrane and the linked ER membrane. To exclude the possibility that the observed difference in diffusion between PtK2 MBP+ and PtK2 MBP− was caused by significant incorporation of the analogs into the ER membrane, we measured the diffusion characteristics of SM in PtK2 cells expressing a chimeric construct (GFP-TM-Lactadherin C2) carrying Lactadherin C2, which contains a phosphatidylserine (PS)-binding domain and, after cell expression, also links organelle membranes such as the ER to the plasma membrane (44). However, we found no indication of a change in the diffusion characteristics of SM in Lactadherin C2-expressing PtK2 cells compared with our control PtK2 cells (Fig. S1). This highlights the specific role of MBP in modifying the diffusion characteristics of the lipids.

**Figure 3:**
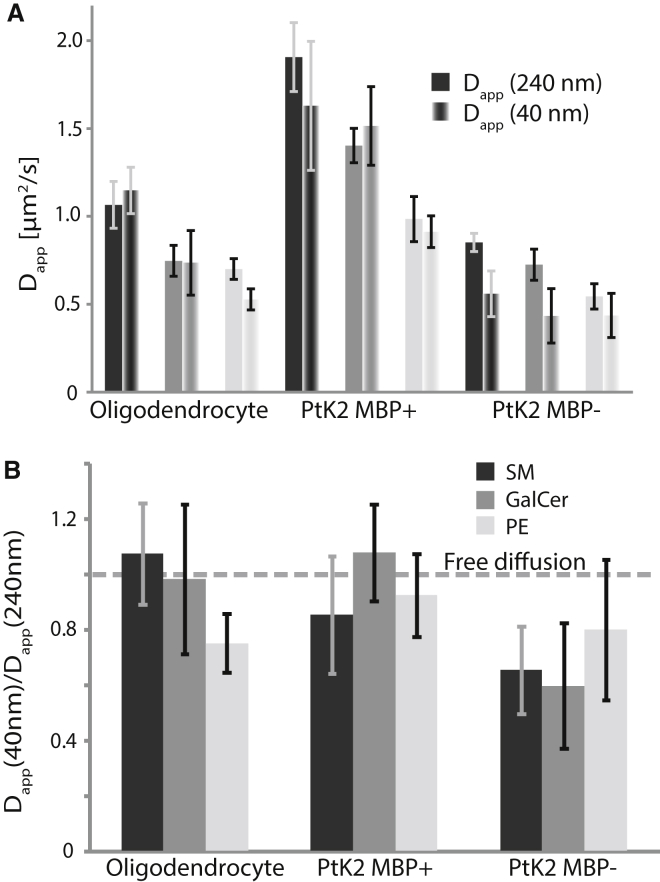
Summary of lipid diffusion data as observed by STED-FCS. (*A*) D_app_ for confocal (*solid columns*, D_app_(240 nm)) and STED (*open columns*, D_app_(40 nm)) recordings of the diffusion of SM (*black*), GalCer (*dark gray*), and PE (*light gray*) analogs in oligodendrocytes, PtK2 cells expressing the MBP-TM-GFP construct (PtK2 MBPþ), and control (nonexpressing) PtK2 (PtK2 MBP) cells, respectively, as labeled. (*B*) Corresponding ratios Δ = D_app_(40 nm)/D_app_(240 nm). Whereas D_app_(240 nm) reveals the macroscopic mobility of the lipids, Δ is an indicator of the degree of hindered diffusion due to transient trapping: the smaller the Δ value, the stronger the trapping, with Δ = 1 indicating free diffusion. Bars show SEM (*n* = 15 cells). All measurements were performed at 37°C.

Differences among diffusion patterns can be made more evident by a direct comparison between D_app_(240 nm) measured with a diffraction-limited observation spot (*d* = 240 nm) and D_app_(40 nm) measured with a subdiffraction observation spot (*d* = 40 nm) (Fig. 3 *A*). Defining the ratio Δ = D_app_(40 nm)/D_app_(240 nm), one can distinguish between free diffusion (Δ ≈ 1) and trapping diffusion (Δ ≪ 1) (Fig. 3 *B*). For all three lipids (PE, SM, and GalCer), diffusion was observed to be approximately free in oligodendrocyte sheets and PtK2 MBP+ domains (Δ > 0.75), with only diffusion of PE in oligodendrocytes being a borderline case (Δ = 0.75). In contrast, as expected from previous STED-FCS data (29, 31, 32), PE diffused approximately freely in control PtK2 MBP− (Δ = 0.80), whereas SM and GalCer underwent clear trapping diffusion in these control cells (Δ = 0.66 and 0.60, respectively).

The overall mobility of the lipids as indicated by D_app_(240 nm) was remarkably large in the MBP+ domains (1.91 ± 0.20 *μ*m^2^/s (SM), 1.40 ± 0.10 *μ*m^2^/s (GalCer), and 0.98 ± 0.13 *μ*m^2^/s (PE)) compared with the PtK2 MBP− controls (0.85 ± 0.05 *μ*m^2^/s (SM), 0.72 ± 0.09 *μ*m^2^/s (GalCer), and 0.54 ± 0.07 *μ*m^2^/s (PE)) and oligodendrocytes (1.07 ± 0.13 *μ*m^2^/s (SM), 0.75 ± 0.09 ± *μ*m^2^/s (GalCer), and 0.70 ± 0.06 *μ*m^2^/s (PE); Fig. 3 *A*). In fact, the PtK2 MBP+ system allowed the highest lipid mobility that we have observed so far with STED-FCS in the plasma membrane of living cells. It is notable that in oligodendrocytes and in PtK2 MBP+ domains, diffusion of SM was the fastest (1.5- to 2-fold faster than that of the other two lipid analogs), followed by GalCer and then PE, eventually correlating with the lipids’ acyl chain lengths (C12 (SM), C13 (GalCer), and C15 (PE)). Yet, we found no difference in the diffusion coefficients of these three analogs in a model membrane bilayer (Fig. S2), indicating an MBP- or oligodendrocyte-specific dependency of the macroscopic diffusion characteristics of the analogs.

**Figure 4:**
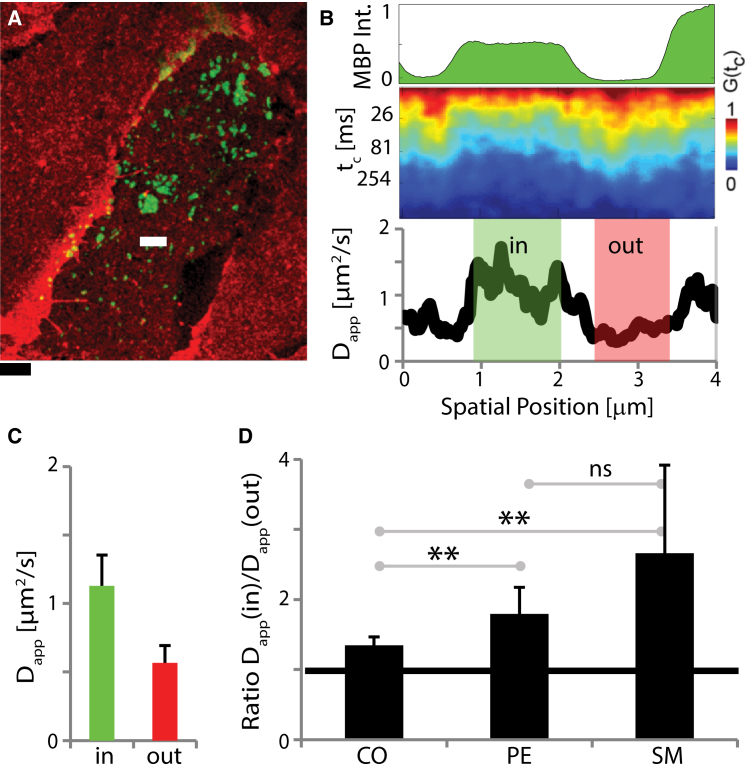
Scanning FCS directly reveals increased lipid mobility in MBP-enriched areas in a single cell. (*A*) Two-color confocal image of the basal membrane of PtK2 cells expressing GFP-TM-MBP (*green*) and incorporating the SM lipid analog (*red*). FCS data were recorded along a line crossing one or more MBP-rich domains (*white line*, length *l* = 4 *μ*m, 60 s, scanning frequency *f* = 4 kHz). Scale bar, 4 *μ*m. (*B*) Representative scanning-FCS data for SM: normalized intensity of GFP-MBP (*top*, *green*), correlation carpet (*middle*, correlation data *G*(*t*_c_) for each pixel of the scan decaying with correlation time *t*_c_ as indicated by the *red to blue* transition), and D_app_ (*bottom*), indicating increased mobility of SM within the domains (in, *green-shaded area*; enhanced GFP signal and faster decays of the correlation data) as compared with outside of the domains (out, *red-shaded area*). (*C*) Average value and standard deviation (*error bars*) of D_app_ of SM inside (in, *green*) and outside (out, *red*) of MBP-rich domains, as determined from 10 independent line scans at different positions. (*D*) Average and standard deviation (*error bars*, 10 independent measurements) of the ratio D_app_(in)/D_app_(out) of lipid mobility inside and outside of the MBP-rich domains for three fluorescent analogs: cholesterol (CO, cholesterol-PEG-KK114), PE, and SM. The increase in mobility when entering the MBP-rich domains was highest for SM, slightly lower for PE, and lowest for the cholesterol analog. All measurements were performed at 37°C. Statistical *p*-test: ^∗∗^significant; ns, nonsignificant. To see this figure in color, go online.

These results were further confirmed by mobility measurements on single cells. Fig. 4 highlights the difference in mobility between MBP-rich domains and the surrounding membrane environment within a single PtK2 cell, as revealed by scanning FCS (45, 46, 47). By scanning the confocal observation spot (*d* = 240 nm) rapidly (frequency *f* = 4 kHz) along a line (4 *μ*m long) crossing the boundary of MBP-rich domains, we recorded FCS data and determined the D_app_ for each pixel (100 pixels over the line), which we then correlated with the GFP signal of the MBP-rich domains. Clearly, the lipid mobility was increased inside the domains, as indicated for SM (Fig. 4, *B* and *C*). Confirming our previous observations, the increase in mobility was higher for SM than for PE (Fig. 4 *D*) after the change from trapping diffusion of SM to fast free diffusion. Interestingly, only a small increase in mobility was observed for a fluorescent cholesterol analog (cholesterol-PEG-KK114, cholesterol labeled with the dye KK114 via a PEG linker). The partitioning of this cholesterol analog into membrane environments of different molecular order was previously determined to be neutral (i.e., with no preference for ordered versus disordered environments) and a diffusion analysis indicated free Brownian motion in PtK2 cells (47). Therefore, this probe most likely reports the average viscosity of the local membrane, which shows only a slight increase in mobility (i.e., decrease in viscosity) in the MBP domains. The stronger difference in diffusion of the SM and PE lipid analogs most likely reflects the reduced protein interactions within MBP domains compared with the crowded rest of the plasma membrane.

The MBP protein may modulate lipid diffusion via distinct mechanisms, ranging from a simple exchange of the structure of the meshwork underlying the plasma membrane to, for example, reordering and sorting of the lipids that are allowed to be in the MBP domains. It was previously observed that oligodendrocytes specifically increase the lipid order (or packing) of membrane sheets by segregation of long-chain lipids into the sheets (8). Therefore, we investigated whether the presence of the MBP meshwork was also able to promote lipid packing and ordering in the domains that formed in the plasma membrane of the PtK2 cells. For this purpose, we expressed MBP fused to the red-emitting fluorescent protein mCherry in live PtK2 cells (mCherry-TM-MBP) to generate and label MBP-enriched domains. In addition, we added the polarity-sensitive membrane dye C-Laurdan, whose emission is relatively blue-shifted (∼440 nm) for more ordered (or packed) membranes containing, for example, more saturated or long-chain lipids, and relatively red-shifted in more disordered membranes (∼490 nm) (48). We used mCherry instead of GFP as a label of MBP to avoid cross talk with the emission of C-Laurdan and any possible energy transfer between the two fluorescent molecules. To observe changes in the emission of C-Laurdan and thus in the lipid membrane order inside and outside the MBP-enriched domains, we applied spectral imaging. This technique allowed us to investigate changes in the emission spectrum of C-Laurdan due to changes in the lipid packing for each image pixel, as highlighted previously (49).

**Figure 5:**
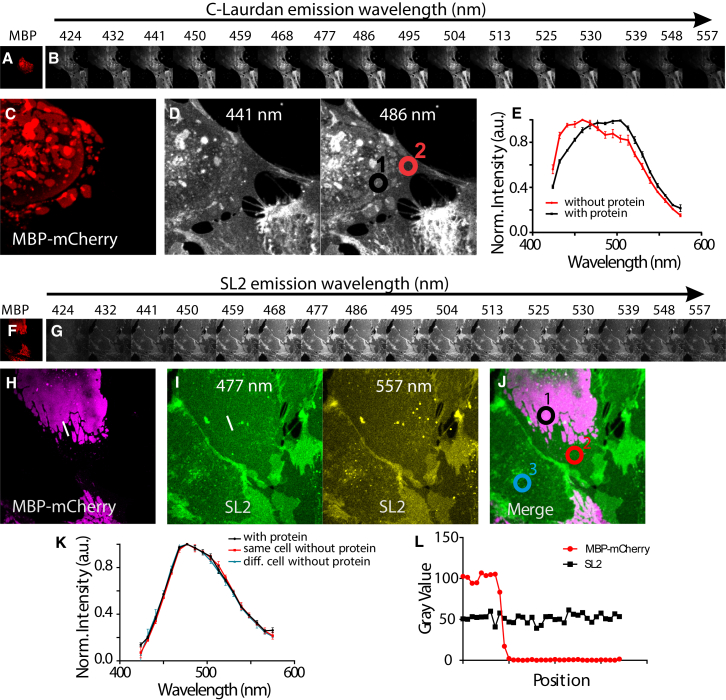
Changes in membrane order in mCherry-TM-MBP-enriched domains of PtK2 cells. (*A*) Large (40 × 40 *μ*m^2^) confocal image of the basal membrane of PtK2 cells expressing mCherry-TM-MBP, indicating the expression and positions of MBP-enriched domains (*red*). (*B*) Images of the same cells for different spectral ranges of the fluorescence emission of C-Laurdan. A slight shift of the fluorescence emission of C-Laurdan toward longer wavelengths is observed within the MBP-enriched domains. (*C* and *D*) Close-up images of (*A*) and (*B*): mCherry fluorescence (*red*, *C*) and emission of C-Laurdan at 441 nm (*D*, *left*) and 486 nm (*D*, *right*). (*E*) Fluorescence emission spectrum of C-Laurdan from areas marked by circles in (*D*): inside (circle 1, *black*) and outside (circle 2, *red*) MBP-enriched domains, revealing a shift of C-Laurdan emission toward longer wavelengths and thus a decrease in membrane order within the domains. (*F*) Large (40 × 40 *μ*m^2^) confocal image of the basal membrane of PtK2 cells expressing mCherry-TM-MBP, indicating the expression and positions of MBP-enriched domains (*red*). (*G*) Images of the same cells for different spectral ranges of the fluorescence emission of SL2. No shift of the fluorescence emission of SL2 is observed within the MBP-enriched domains. (*H–J*) Close-up of a part of the images in (*F*) and (*G*): mCherry fluorescence (*H*, *purple*), emission of SL2 at 477 nm (*I*, *left*), and 557 nm (*I*, *right*), and a merged image (*J*) of mCherry emission (*purple*) and SL2 emission at 477 nm (*gray*). (*K*) Fluorescence emission spectrum of SL2 from areas marked by circles in (*J*): inside (circle 1, *black*) and outside (circle 2, *red*) MBP-enriched domains of the same cell, and on a cell expressing no mCherry-MBP (circle 3, *blue*), revealing no shift of SL2 emission and thus no change in membrane order within the MBP-enriched domains. (*L*) This is further confirmed by plotting the intensity (arbitrarily scaled) of mCherry fluorescence (*red*) and SL2 fluorescence at 477 nm (*black*) along the white line marked in (*I*), crossing the border of the MBP-enriched domain. To see this figure in color, go online.

Fig. 5, *A–E*, shows the results of the spectral imaging of the C-Laurdan emission in correlation to the signal of mCherry marking the MBP-rich domains. The emission of C-Laurdan was more red-shifted, i.e., the membrane was characterized to be more disordered in the MBP-rich domains. This is surprising, since membrane sheets in oligodendrocytes are known to have a very high membrane order (8).

One possible explanation for our result is that a large fraction of C-Laurdan is internalized into cells (50) and thus may potentially also label the internal ER membrane in the close vicinity of the plasma membrane induced by the MBP-enriched domains (Fig. 2 *A*). Therefore, we employed a second probe, SL2. Although this probe is less sensitive than C-Laurdan to lipid packing changes in terms of emission shift, it is useful in investigations of plasma membrane ordering due to its minimal cellular internalization (50). We therefore repeated the spectral imaging experiments using SL2 (Fig. 5, *F–L*). However, we observed no difference in the fluorescence emissions of the plasma-membrane probe SL2 between MBP-rich and -poor regions (Fig. 5 *K*), indicating that MBP did not induce a major change in molecular order. In addition to the spectral shift, the absolute emission intensity of SL2 is also modulated by molecular lipid ordering (51); that is, SL2 is brighter in ordered membrane regions than in disordered membrane regions. When we tested the intensity profile in MBP-rich and -poor regions, we observed no difference (Fig. 5 *L*). Thus, in contrast to membrane sheets in oligodendrocytes, the MBP-induced domains in epithelial PtK2 cells demonstrate no changes in lipid order, indicating that segregation of long-chain lipids into myelin sheets is a process specific to oligodendrocytes.

## Discussion

Previous studies have shown that the fluorescent lipid analogs SM and GalCer undergo trapping diffusion in epithelial cells and fibroblasts (29, 31, 33). Here, we investigated the diffusion of different fluorescent lipid analogs (PE, SM, and GalCer) in oligodendrocytes and found that diffusion in myelin sheets was substantially different from that observed in epithelial cells and fibroblasts; specifically, the mobility was increased and transient arrests were reduced. This strongly suggests that the myelin membrane composition and organization may play an important role in molecular diffusion at the cell surface. We specifically addressed the role of MBP in the dynamic organization of lipids by introducing MBP into PtK2 cells, where MBP forms micrometer-size patches between the ER and the plasma membrane. In these domains, the actin cytoskeleton is effectively replaced by a meshwork of MBP where lipid diffusion is significantly less hindered. Thus, we conclude that MBP is a key modulator of lipid diffusion in myelin.

MBP influences key aspects of the plasma membrane. It depletes proteins from the membrane by forming a diffusion barrier for proteins with large cytoplasmic domains (4, 52) and replaces the cortical network of actin filaments at the cytoplasmic leaflet of the myelin membrane bilayer. In intact cells, transient molecular interactions with slow-moving or immobilized membrane constituents such as proteins have been shown to cause hindered diffusion of lipids, assisted by the cortical actin cytoskeleton (21, 28, 32, 40). These sources of hindered diffusion are missing in MBP-enriched membrane regions and most probably explain the differences in diffusion between these membranes and the plasma membrane of fibroblasts and epithelial cells. Since MBP itself forms a network that underlies the plasma membrane, it may also restrain molecular mobility in the membrane to some extent, for example, by friction or steric effects. Nevertheless, from our findings, we conclude that the extrusion of the actin cytoskeleton and associated membrane proteins seems to be more significant in determining molecular diffusion in the plasma membrane than the interaction between the two-dimensional gel of MBP and the inner leaflet of the plasma membrane.

Another factor that could work against the tendency of faster and less-hindered diffusion in MBP-enriched membrane regions is the formation of a more densely packed and ordered lipid environment. A high degree of lipid packing and order has been observed in membrane sheets of oligodendrocytes (8). However, our experiments with spectral imaging showed that MBP expression and local enrichment did not promote reordering of lipids in the plasma membrane of PtK2 cells. This indicates that segregation of saturated long-chain lipids into membrane sheets is a process specific to oligodendrocytes, possibly requiring the specific lipid composition of myelin-forming cells. On the other hand, the difference in lipid ordering between the myelin sheets of oligodendrocytes and PtK2 MBP+ regions might explain why lipid diffusion is, in general, faster in PtK2 MBP+ than in myelin sheets.

Differences in lipid compositions may also explain why the lipid analogs diffuse much more slowly in membrane sheets of oligodendrocytes as compared with the MBP-enriched domains of the PtK2 cells. The oligodendrocyte-specific enrichment in long-chain, saturated lipids (>C_24_) (6, 8) increases not only the membrane order or packing but also the probability of inner-leaflet coupling, which in turn results in increased friction, viscosity, and lower lipid mobility (53). It would be interesting to study the diffusion of fluorescent lipid analogs with long-chain lipids (>C_24_), but unfortunately, so far we have only been able to efficiently incorporate fluorescent lipid analogs with relatively short-chain fatty acids (<C_20_) into cellular membranes.

Taking into account all of our previous STED-FCS experiments, we can conclude that the MBP-induced domains of PtK2 cells constitute the most fluid cellular-based membrane characterized so far, with free lipid diffusion comparable only to that of model membrane bilayers, but still with a 3-fold lower overall mobility (24, 39). A potential cause of the still slower diffusion in myelin membrane sheets compared with pure model membrane systems may be weak interactions or molecular crowding due to residual proteins or residual actin patches (30).

Here, we did not consider the influences of membrane curvature. This may be an important issue when considering the multilamellar membrane organization of myelin surrounding an axon. However, it is important to note that the curvature of the different myelin layers varies, which may affect how the lipids diffuse. In this study, we wanted to focus on the influence of the MBP proteins on lipid membrane diffusion.

Overall, this work indicates that MBP has major impact on the organization of the plasma membrane by modulating membrane heterogeneities, such as in the supporting actin-cytoskeleton, thereby controlling membrane fluidity and heterogeneity. Clearly, additional mechanisms such as the specific lipid composition and the lamellar membrane structure must be at work in native myelin sheaths.

## Author Contributions

O.S. and D.M.A. designed and conducted experiments, and analyzed the data. V.M. helped with the experiments. A.H. performed the scanning FCS experiments and assisted STED-FCS recordings, E.S. performed the membrane order experiments, and F.S. and E.S. performed the experiments on Lactadherin and GUVs. C.E., M.S., S.W.H., and A.H. designed the research. O.S., D.M.A., C.E., and M.S. wrote the manuscript. All authors contributed in discussing the data, experiments, and manuscript.

## Supporting Citations

References (54, 55, 56, 57) appear in the Supporting Material.

## Supporting Material

Document S1. Supporting Materials and Methods and Figs. S1 and S2

Document S2. Article plus Supporting Material
