# Supplementary material for: Reorganization of Lipid Diffusion by Myelin Basic Protein as Revealed by STED Nanoscopy
Source: Biophys J. 2016 Jun 7;110(11):2441–50. doi: 10.1016/j.bpj.2016.04.047 (PMC4906378; doi:10.1016/j.bpj.2016.04.047)
Supplement: Document S1. Supporting Materials and Methods and Figs. S1 and S2 [file mmc1.pdf]

Biophysical Journal, Volume *110*

Supplemental Information

Reorganization of Lipid Diffusion by Myelin Basic Protein as Revealed by STED Nanoscopy

Olena Steshenko, Débora M. Andrade, Alf Honigmann, Veronika Mueller, Falk Schneider, Erdinc Sezgin, Stefan W. Hell, Mikael Simons, and Christian Eggeling

Supporting Material

MATERIALS AND METHODS

Cell culture

Primary oligodendrocyte culture

Primary cultures of oligodendrocytes were prepared from postnatal day 1 mouse brains as described previously (1). In brief, cellular mixture from trypsinized mouse brains was grown in poly-L-lysine (PLL) coated flasks in Basal Medium Eagle (BME) medium supplemented with 10% horse serum and 100 U/mL each of penicillin and streptomycin. After 7 – 10 days, oligodendroglial progenitors growing on top of a layer of astrocytes were shaken off and cultured further in SuperSato medium on PLL-coated dishes or round coverslips (d = 18 mm) (R.Langenbrinck, Labor- und Medizintechnik, Emmendingen, Germany).

PtK2 epithelial cell line

Cells were grown in flasks with 10 ml of the PtK2-Sato medium. To prevent overgrowth, cells were split once in 6 – 7 days onto a fresh flask. For the imaging, cells were split onto 18 mm coverslips (non-treated with acid and not-covered with PLL), in 1 ml of PtK2-Sato.

PtK2-MBP assay

An assay of the reconstitution of the MBP zipping properties within the epithelial PtK2 cell line was performed as described (2). In brief, the MBP-GFP-TM or MBP-mCherry-TM chimeric construct, containing MBP, transmembrane domain, GFP or mCherry and ER-retention signal, was designed and cloned. Subsequently, PtK2 cells were transfected with this construct and subjected to the further analysis after 18 h post-transfection. Cells, positive to the transfection, were recognised due to the GFP-signal.

PtK2 Lactadherin assay

Transfection, labelling and data acquisition were performed as for cells transfected with MBP-GFP. PtK2 cells were grown on a 25 mm cover slips to a confluency of about 70 %. GFP-TM-Lactadherin C2 domain was transfected using Lipofectamine 3000 (Life Technologies) according to the manufacturer’s protocol. After washing with L15 media, cells were labelled with Atto647N-SM in L15 at a total concentration of 400 µg/mL for 15 minutes at room temperature. After washing with L15 for 3 times, STED-FCS data were acquired in a time window of 1 hour.

Immunolabelling

Primary antibodies used in the study are: rabbit anti-MBP (1:300) (DakoCytomat, Carpinteria, CA), Phalloidin-Rhodamine (F-actin detection) (1:200) (Invitrogen, Munich, Germany), WGA-488 (for lectin detection) (1:200) from (Invitrogen, Munich, Germany). Secondary

fluorophore-conjugated antibodies (Dianova, Hamburg, Germany) were used in 1:200 dilution.

BSA-coupling of fluorescent lipid analogues

To enable incorporation into cellular membranes, lipids analogues were primarily coupled to bovine serum albumin (BSA) as described in (3) with slight modifications. 75 nmol of lipid analogues were first reconstituted in 1:1 chloroform/methanol solution; liquid was further aspirated and lipid films were redissolved in 10 µl of absolute ethanol and vortexed vigorously. Defatted BSA (in Dulbecco's Modified Eagle Medium DMEM without phenol-red, buffered with 10 mM HEPES) was added in equimolar concentration to the lipids. Solution was centrifuged at the maximum speed for 3 min to remove possible aggregates. Supernatant was stored at -20C°.

Incorporation of the lipid analogues

To incorporate lipids into cellular membranes, cells were first washed with 10 mM HEPES-buffered DMEM medium without phenol-red (HDMEM, ice-cold). Next, BSA-lipid complexes, diluted in HDMEM, were added in the appropriate concentrations onto cells in a wet-chamber on ice for 30 min. After incubation, cells were briefly washed with HDMEM and immediately imaged. The fluorescent lipophilic organic dye Atto647N (excitation max at 645 nm, emission max at 670 nm; Atto-Tec, Siegen, Germany) was used as a marker of the incorporated lipids (4-7). Lipids used for the diffusion experiments were as following (5): **PE** – *N*-(Atto647N)-1,2-dihexadecanoyl-*sn*-glycero-3-phosphoethanolamine; **SM** – *N*-(Atto647N)-sphingomyelin; **GalCer –** *N*-(Atto647N)-galactosylsphingosine (Psychosine). Lipids were labelled either at the head group (PE), meaning at the water phase or lipid-water interphase, or via replacement of one the native lipid acyl chains by the short acyl chain carrying a dye (acyl-chain replacement **–** SM, GalC). Throughout the text these lipids are referred simply as PE, SM, GalCer.

Preparation of giant-unilamellar-vesicles (GUVs)

GUVs were prepared by electroformation as described in (Sezgin et al BBA or Garcia-Saez 2009). Briefly, 1 mg/mL DOPC (Avanti Polar Lipids) was spread on platinum wire, evaporated and dipped into 300 mM sucrose solution. GUVs formed during exposure to an electric field of 10 Hz (2V). Lipid analogs, namely Atto647N-DPPE, Atto647N-GalCer and Atto647N-SM were added after formation to a final concentration of about 20, 30 and 100 µg/mL, respectively. GUVs were dropped onto a BSA coated cover slip. The diffusion measurements were performed on the bottom membrane of the vesicles using 640 nm excitation and 650 long pass emission. At least ten different GUVs were measured.

STED-FCS experimental setup

All experiments were performed on a confocal custom-built STED microscope (7) or a STED-modified Abberior Instrument’s Resolft microscope (Abberior Instruments GmbH, Göttingen, Germany) (8). On the first microscope, the confocal unit of the STED nanoscope consisted of an excitation and detection beam path. A fiber-coupled pulsed laser diode

operating at λ_{exc} = 635 nm with a pulse length of 80 ps (LDH-P-635, PicoQuant, Berlin, Germany) was used for excitation of the red fluorescence. After leaving the fiber, the excitation beam was expanded and focused into the sample using an oil immersion objective (HCXPLAPO 100x, NA = 1.4, Leica Microsystems). The fluorescence emitted by the sample was collected by the same objective lens and separated from the excitation light by a custom-designed dichroic mirror (AHF Analysentechnik, Tuebingen, Germany). In the following, the fluorescence was focused onto a multi-mode fiber splitter (Fiber Optic Network Technology, Surrey, Canada). The aperture of the fiber acted as a confocal pinhole of 0.78 of the diameter of the back-projected Airy disk. In addition, the fiber 50:50 split the fluorescence signal, which was then detected by two single-photon counting modules (APD, SPCM-AQR-13-FC, Perkin Elmer Optoelectronics, Fremont, CA). The detector signals were acquired by a single-photon-counting PC card (SPC 830, Becker&Hickl, Berlin, Germany). The confocal setup was extended by integrating a STED laser beam. A modelocked Titanium:Sapphire laser (Ti:Sa, MaiTai, Spectra-Physics, Mountain View, USA) acted as the STED laser emitting sub-picosecond pulses around λ_{STED} = 780 nm with a repetition rate of 80 MHz. The pulses of the STED laser were stretched to 250-350 ps by dispersion in a SF6 glass rod of 50 cm length and a 120 m long polarization maintaining single-mode fiber (PMS, OZ Optics, Ontario, CA). After the fiber, the STED beam passed through a polymeric phase plate (RPC Photonics, Rochester, NY) which introduced a linear helical phase ramp $0 \leq \Phi \leq 2\pi$ across the beam diameter. This wavefront modification gave rise to the doughnut-shaped focal intensity distribution featuring a central intensity zero. The temporal synchronization of the excitation and STED pulses was achieved by triggering the pulses of the excitation laser using the trigger signal from an internal photodiode inside the STED laser and a home-built electronic delay unit, which allowed a manual adjustment of the delay with a temporal resolution of 25 ps. The circular polarization of the STED and excitation laser light in the focal plane was maintained by a combination of $\lambda/2$ and $\lambda/4$ retardation plates in both beam paths (B. Halle, Berlin, Germany).
Integration of a fast scanning unit enabled rapid scanning of the excitation and STED beam across the sample plane (Fig. S1B). A digital galvanometric two mirror scanning unit (Yanus digital scan head, TILL Photonics, Gräfeling, Germany) was used for this purpose. The combination of an achromatic scan lens and a tube lens in a 4f-configuration (f = 50 mm and f = 240 mm, Leica, Wetzlar, Germany) realized a stationary beam position in the back aperture of the objective, preventing peripheral darkening within the focal plane at large scan ranges, such as vignetting. The maximal frequency of the Yanus scanner depended on the scan amplitude and varied between 2 - 6 kHz for scan amplitudes up to 150 µm, respectively. The hardware and data acquisition was controlled by the software ImSpector (http://www.imspector.de/). The synchronization of the beam-scanning for scanning-FCS data acquisition has been described in detail elsewhere (7).

STED-FCS cellular measurements

Day 5 oligodendrocytes and PtK2 cells were grown on standard glass coverslips (diameter 18 mm, no. 1.5 thickness) to a confluence of about 80% at 37C° in a water-saturated atmosphere of 5% CO_2 in the air. Incorporation of the fluorescent lipid analogues (lipid-Atto647N) by cells was accomplished via BSA coupling. Measurements were performed at 37C° in HDMEM.

As detailed previously (5, 7, 8), we ensured a non-detectable (at least by STED-FCS) influence by the dye label, and that the observation times were given by the focal transit times and not shortened due to photobleaching (by measuring at low enough excitation intensities), and that biasing effects by the excitation or STED light due to photobleaching, heating or other (non-linear) effects and diffusion of non-integrated lipids (or dye tags) could

be excluded. Also, we can exclude a significant amount of immobilized lipid analogues. Such fraction would have become visible at the beginning of an FCS measurement by a decrease in the overall fluorescence intensity, which we have not detected. In addition, previous FRAP (Fluorescence Recovery After Photobleaching) experiments on SM and PE in PtK2 cells showed an immobile fraction of < 5% (5, 7, 8).

We assessed the dynamics of fluorescent lipid analogues by placing the focused co-centred excitation and STED beams on random positions at the plasma membrane and completed all measurements before any significant morphological changes in the cell could occur. All measurements were carried out during 10 s, providing a correlation time longer than two orders of magnitude times the typical transient time of the labelled lipids through the confocal observation area. The calculated apparent diffusion coefficient for each given effective beam diameter resulted from the average of measurements performed in different cells. For each cell analysed, the apparent diffusion coefficient for each given effective beam diameter resulted from the average of at least 2 and up to 8 repetitions of the STED-FCS measurement at the given cell. For each given effective beam diameter, the averaged values obtained from individual cells were then averaged so that the correspondent standard error of the mean reflects the variance among cells.

STED-FCS analysis

We fitted all correlation data $G(t_c)$ with a model for two-dimensional diffusion,

$$G(t_c) = DC + \frac{1}{N}\frac{1}{1+t_c/t_D} + Texp(-\frac{t_c}{t_T})$$

Where t_c is the correlation time, N is the average number of fluorescent particles in the observation spot, t_D is the average transit time of the fluorescent molecules through the observation spot, and T and t_T are the amplitude and characteristic correlation time accounting for the population kinetics of the fluorescent molecules' dark triplet state. Correlation data were recorded with lipid concentrations resulting in a temporal average of particle number $N \approx 0.5$–10 fluorescent particles for the highest STED depletion and $N \approx 10$–200 fluorescent particles for confocal recordings. The correspondent apparent diffusion coefficient D was calculated based on the knowledge of the observation diameter d of the observation spot, provided by the calibration of the system (see below), $D = d^2/(8 \cdot t_D \cdot ln(2))$. To obtain a final value of D for each measurement condition (different STED powers or diameters d and different lipids), values determined from measurements taken on the same cell were first averaged and then a final average calculated from averaging all average values of all (n) individual cells measured in this condition. We usually recorded at least 5 curves per cell for n = 10-20 cells. The standard error of the mean (s.e.m.) was calculated from the deviations in-between cells.

STED-FCS calibration

Supported lipid bilayers (SLBs) were used to calibrate the STED-FCS setup. The SLBs were prepared based on a procedure described elsewhere (9). Briefly, the lipid DOPC (1,2-dioleoyl-sn-glycero-3-phosphocholine, Avanti, Alabaster, AL) and a fluorescent lipid analogue (DPPE-Atto647N, Atto-Tec, Siegen, Germany) were mixed in organic solvents (Chloroform/MeOH 1:1) at a lipid concentration of 1 mg/ml. The ratio of labelled lipids per non-labelled ones was approximately 1:10000. 50 ml of such solution were dropped onto a piranha-cleaned standard microscope cover glass (diameter 22 mm, no. 1.5 thickness) and spin-coated at 60 Hz for about one minute. The cover glass was then placed in a microscopy

chamber and subsequently the dry thin lipid film was rehydrated with 500 µl buffer solution (150 mM NaCl, 10 mM HEPES). Such bilayers were stable for several hours.

Calibration of the diameter $d(P_{STED})$ of the effective focal fluorescence spots formed by a certain STED power P_{STED} was performed by STED-FCS measurements of fluorescent lipid analogues in supported lipid bilayers (SLBs), for that such lipid bilayers provide a two-dimensional free diffusing system of molecules, labelled with the same fluorophore used in our cellular experiments. The confocal observation diameters being determined by fluorescent beads ($d(P_{STED} = 0) \approx 240$nm), the other effective diameters $d(P_{STED} \neq 0)$ can be calculated by performing STED-FCS measurements on supported lipid bilayers and using the relation:

$$\frac{d(P_{STED} = 0)}{d(P_{STED})} = \sqrt{\frac{t_D(P_{STED} = 0)}{t_D(P_{STED})}}$$

where t_D stands for the average transient times correspondent to each given P_{STED}. The relation above stems from the fact that the lipids in SLBs undergo two-dimensional free diffusion, so that the diffusion time scales proportionally with the diffusion area.

Spectral Imaging

Spectral imaging of the different membrane samples were performed on a Zeiss LSM 780 confocal microscope equipped with a 32-channel GaAsP detector array, as highlighted previously (10). Laser light at 405 nm was selected for fluorescence excitation of C-Laurdan and SL2. The lambda detection range was set between 415 nm and 691 nm. Despite the fact that wavelength intervals of down to 4 nm can be chosen for the individual detection channels, we have set these intervals to 8.9 nm, which allowed the simultaneous coverage of the whole spectrum with the 32 detection channels. The images were saved in .lsm file format.

The spectra for each image pixel were obtained from the intensity values of the 32 different detection channels by using the ImageJ plug-in “Stacks-T functions-Intensity vs. Time Monitor”, which has been described in detail previously (10) and can be downloaded at https://github.com/dwaithe/GP-plugin. We usually applied the plugin only on pixels within a region of interest of the acquired images. Background signal was determined by applying the same plugin on a dark region (of the same size) of the image, and subtracted from the signal from the region of interest.

Confocal Microscopy

Confocal microscopic images were acquired with a Leica DMIRE2 microscope and a Leica TCS SP2 AOBS confocal laser scanning setup (Leica Microsystems, Mannheim, Germany). 40X NA 1.25 or 63X NA 1.4 oil plan-apochromat objectives (Leica Microsystems, Mannheim, Germany) were used for image acquisition.

Image Processing and Statistical Analysis

Images were processed and analysed with the public domain Java-based image processing software ImageJ (created by Rasband, W.S., National Institutes of Health, Bethesda,

Maryland, USA), or with a MBF collection of plugins for ImageJ ("MBF ImageJ for Microscopy").

Statistical analysis was performed using MS Office Excel and GraphPad Prism software; unpaired student t-test was used as a statistical test.

SUPPORTING REFERENCES

1. Fitzner D, Schneider A, Kippert A, Mobius W, Willig KI, Hell SW, Bunt G, Gaus K, Simons M. Myelin basic protein-dependent plasma membrane reorganization in the formation of myelin. EMBO J 2006;25(5037-5048):5037-5048.
2. Aggarwal S, Snaidero N, Pahler G, Frey S, Sanchez P, Zweckstetter M, Janshoff A, Schneider A, Weil MT, Schaap IA, Gorlich D, Simons M. Myelin membrane assembly is driven by a phase transition of myelin basic proteins into a cohesive protein meshwork. PLoS Biol 2013;11(6):e1001577.
3. Martin OC, Pagano RC. Internalization and sorting of a fluorescent analogue of glucosylceramide to the Golgi apparatus of human skin fibroblasts: utilization of endocytic and nonendocytic transport mechanisms. J Cell Biol 1994;125:769-781.
4. Eggeling C, Ringemann C, Medda R, Schwarzmann G, Sandhoff K, Polyakova S, Belov VN, Hein B, von Middendorff C, Schonle A, Hell SW. Direct observation of the nanoscale dynamics of membrane lipids in a living cell. Nature 2009;457:1159-1162.
5. Mueller V, Ringemann C, Honigmann A, Schwarzmann G, Medda R, Leutenegger M, Polyakova S, Belov VN, Hell SW, Eggeling C. STED nanoscopy reveals molecular details of cholesterol- and cytoskeleton-modulated lipid interactions in living cells. Biophys J 2011;101:1651-1660.
6. Mueller V, Honigmann A, Ringemann C, Medda R, Schwarzmann G, Eggeling C. FCS in STED Microscopy: Studying the Nanoscale of Lipid Membrane Dynamics. In: Tetin SY, editor. Methods Enzymol. Burlington: Academic Press: Elsevier; 2013. p. 1-38.
7. Honigmann A, Mueller V, Ta H, Schoenle A, Sezgin E, Hell SW, Eggeling C. Scanning STED-FCS reveals spatio-temporal heterogeneity of lipid interaction in the plasma membrane of living cells. Nature Communications 2014;5:5412.
8. Clausen MP, Galiani S, Bernardino de la Serna J, Fritzsche M, Chojnacki J, Gehmlich K, Lagerholm BC, Eggeling C. Pathways to optical STED microscopy. NanoBioImaging 2013;1(1):1-12.
9. Chiantia S, Kahya N, Ries J, Schwille P. Effects of Ceramide on Liquid-Ordered Domains Investigated by Simultaneous AFM and FCS. Biophys J 2006;90:4500–4508.
10. Sezgin E, Waithe D, Bernardino de la Serna J, Eggeling C. Spectral Imaging to Measure Heterogeneity in Membrane Lipid Packing. Chemphyschem 2015;16(7):1387-1394.

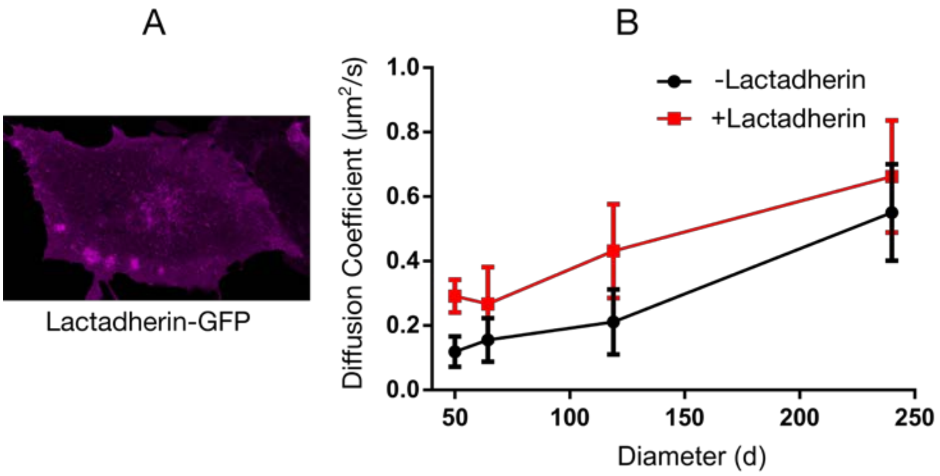

Supplementary Figure 1: Diffusion of SM with and without Lactadherin C2. (**A**) Exemplary image of the bottom membrane of GFP-TM-Lactadherin C2 domain transfected Ptk2 cells (purple: GFP signal). (**B**) STED-FCS data of SM with (+) and without (-) Lactadherin C2, indicating slightly faster diffusion in the case of GFP-TM-Lactadherin expression, but more importantly trapping in both cases. Error bars correspond to the standard deviation determined from two independent data sets including several cells.

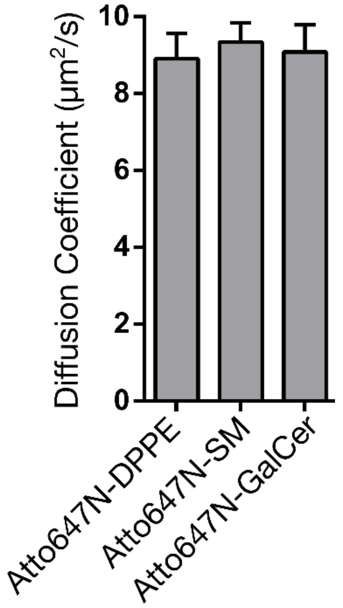

Supplementary Figure 2: Diffusion coefficient determined from confocal FCS measurements of Atto647N-labeled PE, SM and GalCer in GUVs, indicating statistically similar diffusion coefficients. Error bars represent standard deviation determined from 10 independent measurements.
