# Supplementary material for: Reorganization of Lipid Diffusion by Myelin Basic Protein as Revealed by STED Nanoscopy
Source: Biophys J. 2016 Jun 7;110(11):2441–50. doi: 10.1016/j.bpj.2016.04.047 (PMC4906378; doi:10.1016/j.bpj.2016.04.047)
Supplement: Document S2. Article plus Supporting Material [file mmc2.pdf]

Biophysical Journal
Article

Biophysical Society

Reorganization of Lipid Diffusion by Myelin Basic Protein as Revealed by STED Nanoscopy

Olena Steshenko,[1] Débora M. Andrade,[2,3] Alf Honigmann,[2,4] Veronika Mueller,[2] Falk Schneider,[5] Erdinc Sezgin,[5] Stefan W. Hell,[2] Mikael Simons,[1,6,*] and Christian Eggeling[2,5,*]

[1]Cellular Neuroscience, Max Planck Institute of Experimental Medicine, Göttingen, Germany; [2]Department of Nanobiophotonics, Max Planck Institute for Biophysical Chemistry, Göttingen, Germany; [3]Centre for Neural Circuits and Behaviour, University of Oxford, Oxford, United Kingdom; [4]Max Planck Institute of Molecular Cell Biology and Genetics, Dresden, Germany; [5]MRC Human Immunology Unit, Weatherall Institute of Molecular Medicine, University of Oxford, Oxford, United Kingdom; and [6]Institute of Neuronal Cell Biology, Technical University Munich, Munich, Germany

ABSTRACT Myelin is a multilayered membrane that ensheathes axonal fibers in the vertebrate nervous system, allowing fast propagation of nerve action potentials. It contains densely packed lipids, lacks an actin-based cytocortex, and requires myelin basic protein (MBP) as its major structural component. This protein is the basic constituent of the proteinaceous meshwork that is localized between adjacent cytoplasmic membranes of the myelin sheath. Yet, it is not clear how MBP influences the organization and dynamics of the lipid constituents of myelin. Here, we used optical stimulated emission depletion super-resolution microscopy in combination with fluorescence correlation spectroscopy to assess the characteristics of diffusion of different fluorescent lipid analogs in myelin membrane sheets of cultured oligodendrocytes and in micrometer-sized domains that were induced by MBP in live epithelial PtK2 cells. Lipid diffusion was significantly faster and less anomalous both in oligodendrocytes and inside the MBP-rich domains of PtK2 cells compared with undisturbed live PtK2 cells. Our data show that MBP reorganizes lipid diffusion, possibly by preventing the buildup of an actin-based cytocortex and by preventing most membrane proteins from entering the myelin sheath region. Yet, in contrast to myelin sheets in oligodendrocytes, the MBP-induced domains in epithelial PtK2 cells demonstrate no change in lipid order, indicating that segregation of long-chain lipids into myelin sheets is a process specific to oligodendrocytes.

INTRODUCTION

Myelin is a highly specialized membrane that forms a continuous, multilayered stack of tightly packed membrane, ensheathing the axons in the central and peripheral nervous systems (1,2). In contrast to most plasma membranes, the myelin membrane contains a high proportion of lipids, which account for 80% of its dry weight (3,4). Remarkably, the myelin membrane is enriched in cholesterol, plasmalogens, and galactosylceramide lipid species, with a high fraction of lipids with long-chain fatty acids (5–7). The self-segregation of these very-long-chain glycosphingolipids from other, shorter lipids may be a driving factor in the generation of the highly ordered myelin membrane (8). This high content of tightly packed lipids is essential for the electrical insulating properties of the myelin membrane that are required to increase the propagation speed of the impulses along myelinated fibers (1,9).

The key molecule that organizes the structure of myelin membrane sheets is myelin basic protein (MBP) (10,11). This intrinsically disordered polypeptide chain has a strong basic character and polymerizes into a cohesive mesh-like protein network when bound to the inner leaflet of membrane bilayers (12–14). Within the cytoplasmic space of the compact myelin sheath, MBP forms a size-selective barrier that prevents diffusion of most cytosolic and membrane proteins into the compact myelin sheath region (4,15). In addition to its specific lipid composition and low amount of proteins, the compact myelin sheath also lacks an actin-based cytocortex (4,16,17).

Due to these characteristics, as well as its abundance and ordered laminar structure, myelin has served as a model membrane for many biochemists and biophysicists (18,19). However, it is not yet clear how the large amount of structural information on myelin relates to the general principles of membrane dynamics and thus membrane bioactivity.

Submitted October 14, 2015, and accepted for publication April 25, 2016.

*Correspondence: simons@em.mpg.de or christian.eggeling@rdm.ox.ac.uk

Olena Steshenko and Débora M. Andrade contributed equally to this work.

Editor: Katharina Gaus.

http://dx.doi.org/10.1016/j.bpj.2016.04.047

© 2016 Biophysical Society

This is an open access article under the CC BY license (http://creativecommons.org/licenses/by/4.0/).

CrossMark

Over the past few years, investigators have refined the basic model of membrane structure organized as homogeneous fluid mosaics (20) by including the concept of heterogeneity. Lipid-lipid and lipid-protein interactions, protein oligomerization, and the underlying cortical actin cytoskeleton are major driving forces that compartmentalize the plasma membrane (21–23). Prominent examples include lipid microdomains, caveolae, coated pits, and actin cytoskeleton-induced subcompartments (24–28). Due to this patchwork organization of the plasma membrane, diffusion of molecules is in many cases not free, but anomalous (21,24,29–32). For example, interactions with less mobile entities such as proteins lead to transient slowdowns in diffusion (trapping) (22,29,31,33), whereas actin-induced compartments result in temporary confinements within 30–300 nm compartments (hop diffusion) (24,30,34,35). All of these structural and diffusional heterogeneities rule the bioactivity of membrane molecules (36).

Recent studies have investigated plasma membrane dynamics by exploiting the nanoscale spatial resolution provided by optical stimulated emission depletion (STED) microscopy (or nanoscopy) (37) in conjunction with fluorescence correlation spectroscopy (STED-FCS) (29,38) to observe anomalous diffusion of membrane molecules, such as the transient binding of lipids to other membrane constituents (31,32,39,40). STED-FCS enabled investigators to make a direct distinction between free and anomalous diffusion (29). These studies demonstrated that the diffusion of phosphoglycerolipids without hydroxyl-containing headgroups was relatively free, showing only weak interactions within the membrane. The strongest confinements among the tested lipids were observed for sphingolipid analogs that showed cholesterol-assisted and cytoskeleton-dependent transient trapping, most probably due to transient binding to immobile or slow-moving proteins (29,31,32).

Taking into account the peculiar organization of the myelin membrane, in this work we used STED-FCS in combination with fluorescent lipid analogs to address the question of how MBP organizes heterogeneity in lipid diffusion. We observed significantly faster and less anomalous lipid diffusion both in oligodendrocytes (of which MBP is a ubiquitous component) and in MBP-rich domains of MBP-transfected epithelial PtK2 cells. Using two different polarity-sensitive fluorescent lipid reporters, we further tested whether these MBP-rich domains are characterized by a higher molecular lipid order, as was previously reported for oligodendrocytes. However, our data showed no difference in order between inside and outside of the domains.

MATERIALS AND METHODS

Preparation of oligodendrocytes and epithelial PtK2 cells on glass coverslips, incorporation of the fluorescent lipid analogs into the plasma membrane of the living cells via bovine serum albumin complexes, the PtK2-MBP assay, immunolabeling and confocal imaging details, STED-FCS measurement conditions and procedures, the STED-FCS setup and its calibration, the spectral imaging setup, and the FCS analysis are described in the Supporting Materials and Methods.

RESULTS

To study lipid diffusion in myelin, we first used primary cultures of oligodendrocytes as an experimental system. When cultured on glass coverslips, oligodendrocytes form large myelin membrane sheets (Fig. 1). This system satisfies many of the essential requirements for studying the structure of the myelin membrane. First, the membrane sheets resemble in vivo compact myelin in their lipid and protein compositions. The main components of compact myelin localize to membrane sheets, such as the MBP, while most other proteins are excluded, as shown by staining for sialic acid and N-acetylglucosaminyl protein residues (wheat germ agglutinin (WGA); Fig. 1 *A*). In addition, as shown previously (16,17), staining for actin revealed no distinct actin cytoskeleton network within the sheets (Fig. 1 *A*). As the cortical actin network is one of the main modulators of molecular diffusion in the plasma membrane, we measured lipid mobility in the membrane sheets. For this purpose, we incorporated different lipid analogs labeled with the lipophilic organic dye Atto647N into the plasma membrane of living oligodendrocytes by incubation with lipid-bovine serum albumin complexes, and monitored their diffusion pattern using STED-FCS. All measurements reported here were performed in living cells at 37°C. Other than the unexpected finding in previous studies that Atto647N-labeled saturated lipid analogs partitioned into the liquid-disordered (instead of the liquid-ordered) phase in model membranes (31,41), this label has not been found to have any notable effect on the interactions of lipids in live cells, at least as observed by STED-FCS (29,31,32). We chose to use fluorescent lipid analogs of saturated phosphatidylethanolamine (referred to as phosphoethanolamine or simply PE, acyl chain length C15, label at headgroup) and saturated sphingomyelin (SM, C12, labeling via acyl-chain replacement) for our diffusion studies because their behavior in epithelial cells and fibroblasts has been shown to be significantly distinguishable (29,31,32). In addition, we chose a fluorescent analog of a highly abundant myelin lipid, galactosylceramide (GalCer, C13, labeling via acyl-chain replacement).

STED-FCS allows one to determine the apparent diffusion coefficient, D_{app}, for different sizes, i.e., different diameters, d, of the observation spot, typically $d = 40$–240 nm (31). Although D_{app} values determined from FCS data of the (diffraction-limited) confocal observation spot ($d = 240$ nm) only report on the average (relatively long-scale) mobility of the lipids, the observation and comparison of D_{app} values for different sizes of the observation spot highlight characteristics of the diffusion pattern

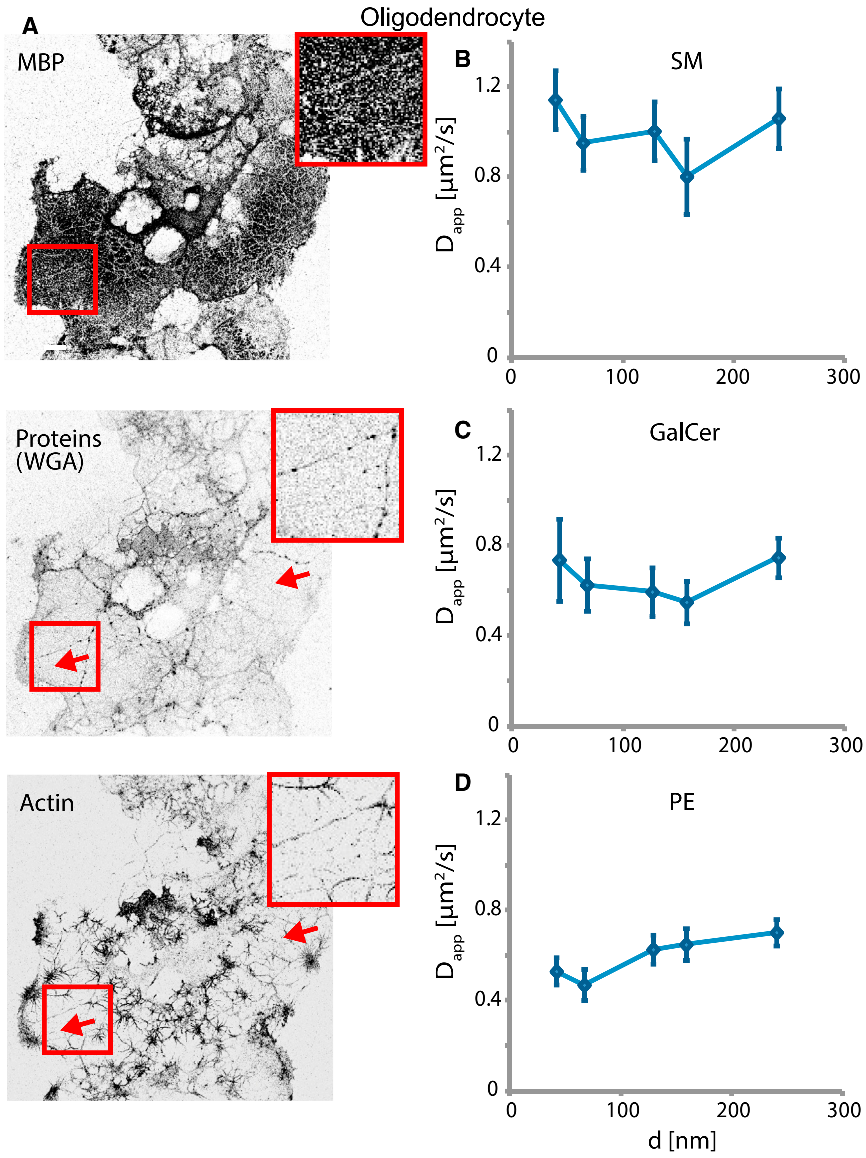

FIGURE 1 Lipids diffuse approximately freely in oligodendrocyte membrane sheets. (*A*) Confocal fluorescence images (50 × 50 μm^2, inverted grayscale from *white* (low signal) to *black* (high signal), contrast adapted between the different images to highlight differences) of cultured oligodendrocytes fixed with 4% paraformaldehyde and 0.25% glutaraldehyde on day 5. The images show cell-surface fluorescence staining for MBP (*top*), sialic acid and N-acetylglucosaminyl residues with WGA-Alexa488 conjugate for general protein staining (*middle*), and after permeabilization and fluorescence immunostaining for filamentous actin with rhodamine-phalloidin (*bottom*), indicating that proteins and actin were depleted from the membrane sheet areas (representatively indicated by the *red arrows* and *red flanked insets*, which show zoom-ins of the areas marked with a *red box*). (*B–D*) STED-FCS analysis, showing the dependence of D_{app} on the diameter *d* of the microscope's observation spot for the fluorescent lipid analogs SM (*top*), GalCer (*middle*), and PE (*bottom*) in the myelin sheets at 37°C. The roughly constant values indicate approximately free diffusion. The plots show mean ± standard error of the mean (SEM) (n = 15 cells). To see this figure in color, go online.

(29,31,42). The independence of the observed D_{app} with respect to the size of the observation spot is indicative of free diffusion (31). In this case, diffusion is not hindered (at least on the spatial scales investigated, i.e., $d > 40$ nm) and is only limited by the characteristic viscosity of the medium in which the molecules diffuse. Conversely, when D_{app} is observed to increase as the size of the observation spot is increased, the molecules experience trapping, i.e., diffusion is transiently slowed down or the molecules are arrested on small spatial scales due to, for example, transient interactions with slower-moving or immobilized binding partners (31,42). For small observation spots, the transit time of trapped molecules is much more dominated by the trapping time and thus leads to a decrease of the measured D_{app} (in contrast to the real instant diffusion coefficient, which stays the same in between the traps). This should not be confused with findings from single-particle tracking (SPT) experiments, where trapping usually results in reduced long-scale (or macroscopic) diffusion coefficients compared with the large (fast) real instant or microscopic diffusion coefficient in between traps as measured on short spatial scales (or for small time steps). These two techniques (STED-FCS and SPT) indeed assess confined diffusion differently, as highlighted by the fact that hopping diffusion and trapping diffusion are indistinguishable from the perspective of SPT, whereas in STED-FCS these two modalities of anomalous diffusion are observed fundamentally differently (32,43).

Our STED-FCS recordings on oligodendrocytes revealed that all lipid analogs investigated here diffused approximately freely in membrane sheets, with $D_{app} \approx$ 0.70–1.0 μm^2/s, the same range as the average mobility that was previously reported in conventional confocal FCS recordings of a Bodipy-labeled lipid analog (8) (Fig. 1, *B–D*). Although previous STED-FCS studies showed that

PE diffused approximately freely in the plasma membrane of epithelial cells and fibroblasts, conversely, they also showed that SM and GalCer underwent trapping diffusion in those cells (29,31,32).

These findings pinpoint the peculiar nature of myelin membrane sheets. We previously showed that MBP expression as a chimeric construct (green fluorescent protein (GFP) labeled (GFP-TM-MBP)) generates connections between the membrane of the endoplasmic reticulum (ER) and the plasma membrane in epithelial PtK2 cells (Fig. 2 *A*) (13). These micrometer-sized, protein-poor domains resemble compact myelin in terms of protein composition (enriched in MBP and deprived in N-acetylglucosaminyl protein residues) and also lack a cortical actin cytoskeleton (Fig. 2 *B*). To investigate the specific role of MBP in modulating lipid diffusion in membranes, we took advantage of this system of GFP-TM-MBP-transfected PtK2 cells and observed diffusion via STED-FCS inside and outside of MBP domains (Fig. 2, *C–E*). We found that all three lipid analogs (SM, GalCer, and PE) diffused at least twice as fast within the domains (PtK2 MBP+) as compared with the control (nonexpressing) PtK2 cells (PtK2 MBP−). Moreover, diffusion of all lipid analogs appeared to be free within MBP domains, which is in contrast to the diffusion of SM and GalCer in the control PtK2 MBP− cells.

We have to note that we cannot distinguish between lipid diffusion in the outer and inner leaflets of the plasma membrane, or, in the case of the PtK2 MBP+ domains, between diffusion of the lipid analogs in the plasma membrane and the linked ER membrane. To exclude the possibility that the observed difference in diffusion between PtK2 MBP+ and PtK2 MBP− was caused by significant incorporation of the analogs into the ER membrane, we measured the diffusion characteristics of SM in PtK2 cells expressing a

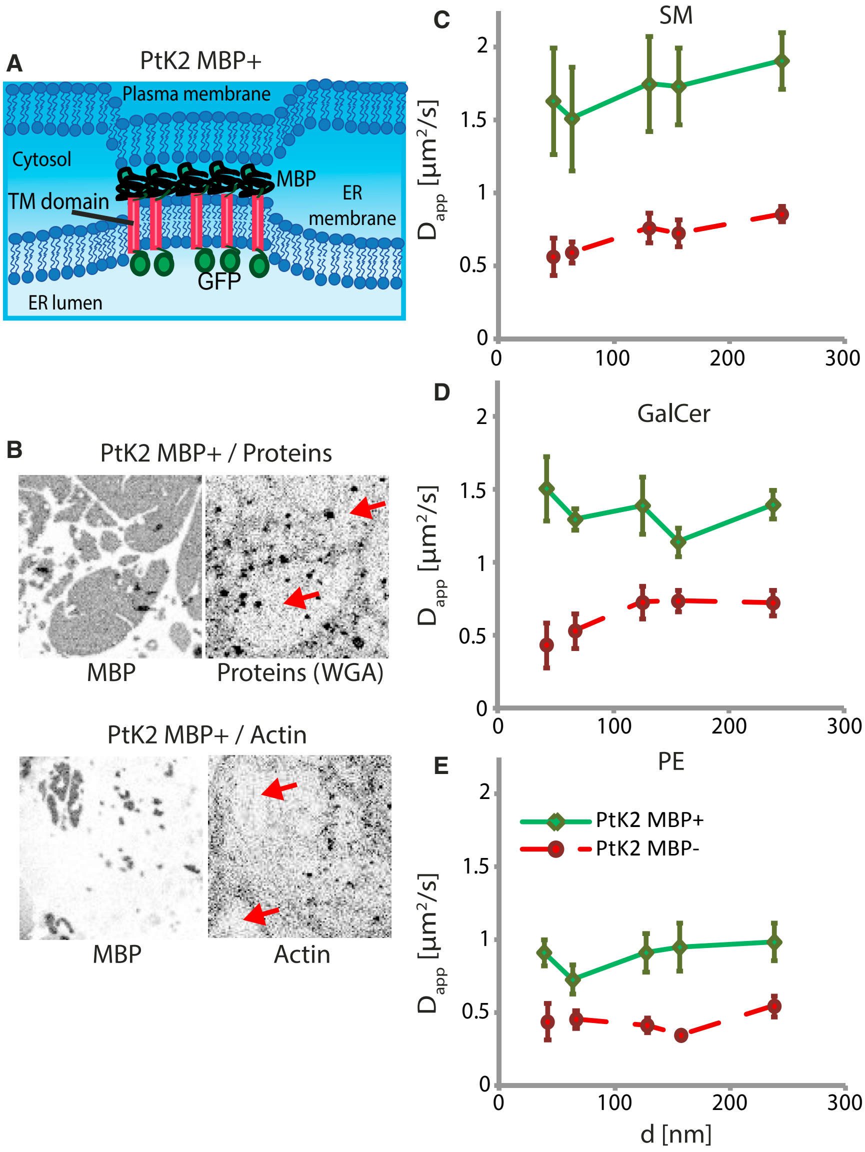

FIGURE 2 The formation of MBP-mediated membrane patches alters lipid diffusion in PtK2 cells. (*A*) Schematic drawing of MBP-mediated membrane domains: upon expression in PtK2 cells, the MBP-expressing chimeric fluorescent protein GFP-TM-MBP forms ER-plasma membrane patches that are depleted of glycoproteins and cortical actin cytoskeleton. (*B*) Confocal fluorescence images (6 × 6 μm^2, inverted grayscale from *white* (low signal) to *black* (high signal), contrast adapted between the different images to highlight differences) of cultured and GFP-TM-MBP transfected epithelial PtK2 cells (PtK2 MBP+) fixed with 4% paraformaldehyde and 0.25% glutaraldehyde, at 16 h after transfection. The panel shows fluorescence immunostaining for sialic acid and N-acetylglucosaminyl protein residues with WGA-Alexa488 (panel labeled with proteins (WGA)) and filamentous actin with rhodamine-phalloidin label (panel labeled with actin), and the respective GFP-TM-MBP images (panels labeled with MBP). As for membrane sheets, proteins and F-actin were depleted from the MBP-mediated membrane patches (representatively indicated by the *red arrows*). (*C–E*) STED-FCS analysis, showing the dependence of D_{app} on the diameter *d* of the microscope's observation spot for the fluorescent lipid analogs SM (*C*), GalCer (*D*), and PE (*E*) in PtK2 MBP+ (*green*) and PtK2 MBP− (*red dotted*) cells. Bars show SEM (n = 15 cells). All measurements were performed at 37°C. To see this figure in color, go online.

chimeric construct (GFP-TM-Lactadherin C2) carrying Lactadherin C2, which contains a phosphatidylserine (PS)-binding domain and, after cell expression, also links organelle membranes such as the ER to the plasma membrane (44). However, we found no indication of a change in the diffusion characteristics of SM in Lactadherin C2-expressing PtK2 cells compared with our control PtK2 cells (Fig. S1). This highlights the specific role of MBP in modifying the diffusion characteristics of the lipids.

Differences among diffusion patterns can be made more evident by a direct comparison between D_{app}(240 nm) measured with a diffraction-limited observation spot (d = 240 nm) and D_{app}(40 nm) measured with a subdiffraction observation spot (d = 40 nm) (Fig. 3 *A*). Defining the ratio $\Delta = D_{app}(40\ nm)/D_{app}(240\ nm)$, one can distinguish between free diffusion ($\Delta \approx 1$) and trapping diffusion ($\Delta << 1$) (Fig. 3 *B*). For all three lipids (PE, SM, and GalCer), diffusion was observed to be approximately free in oligodendrocyte sheets and PtK2 MBP+ domains ($\Delta > 0.75$), with only diffusion of PE in oligodendrocytes being a borderline case ($\Delta = 0.75$). In contrast, as expected from previous STED-FCS data (29,31,32), PE diffused approximately freely in control PtK2 MBP− ($\Delta = 0.80$), whereas SM and GalCer underwent clear trapping diffusion in these control cells ($\Delta = 0.66$ and 0.60, respectively).

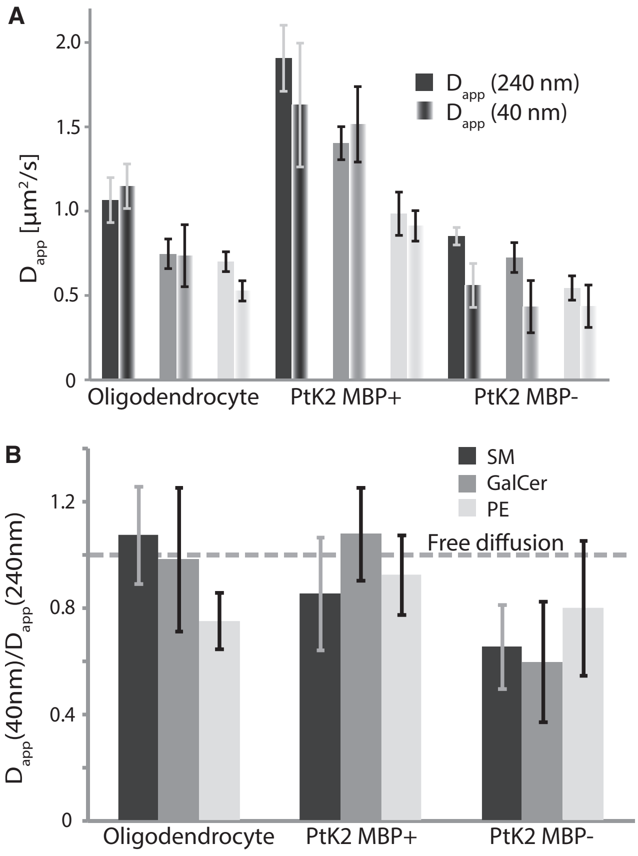

FIGURE 3 Summary of lipid diffusion data as observed by STED-FCS. (*A*) D_{app} for confocal (*solid columns*, D_{app}(240 nm)) and STED (*open columns*, D_{app}(40 nm)) recordings of the diffusion of SM (*black*), GalCer (*dark gray*), and PE (*light gray*) analogs in oligodendrocytes, PtK2 cells expressing the MBP-TM-GFP construct (PtK2 MBPþ), and control (nonexpressing) PtK2 (PtK2 MBP) cells, respectively, as labeled. (*B*) Corresponding ratios $\Delta = D_{app}(40\ nm)/D_{app}(240\ nm)$. Whereas D_{app}(240 nm) reveals the macroscopic mobility of the lipids, Δ is an indicator of the degree of hindered diffusion due to transient trapping: the smaller the Δ value, the stronger the trapping, with $\Delta = 1$ indicating free diffusion. Bars show SEM (n = 15 cells). All measurements were performed at 37°C.

The overall mobility of the lipids as indicated by D_{app}(240 nm) was remarkably large in the MBP+ domains (1.91 ± 0.20 $\mu m^2/s$ (SM), 1.40 ± 0.10 $\mu m^2/s$ (GalCer), and 0.98 ± 0.13 $\mu m^2/s$ (PE)) compared with the PtK2 MBP− controls (0.85 ± 0.05 $\mu m^2/s$ (SM), 0.72 ± 0.09 $\mu m^2/s$ (GalCer), and 0.54 ± 0.07 $\mu m^2/s$ (PE)) and oligodendrocytes (1.07 ± 0.13 $\mu m^2/s$ (SM), 0.75 ± 0.09 ± $\mu m^2/s$ (GalCer), and 0.70 ± 0.06 $\mu m^2/s$ (PE); Fig. 3 *A*). In fact, the PtK2 MBP+ system allowed the highest lipid mobility that we have observed so far with STED-FCS in the plasma membrane of living cells. It is notable that in oligodendrocytes and in PtK2 MBP+ domains, diffusion of SM was the fastest (1.5- to 2-fold faster than that of the other two lipid analogs), followed by GalCer and then PE, eventually correlating with the lipids' acyl chain lengths (C12 (SM), C13 (GalCer), and C15 (PE)). Yet, we found no difference in the diffusion coefficients of these three analogs in a model membrane bilayer (Fig. S2), indicating an MBP- or oligodendrocyte-specific dependency of the macroscopic diffusion characteristics of the analogs.

These results were further confirmed by mobility measurements on single cells. Fig. 4 highlights the difference in mobility between MBP-rich domains and the surrounding membrane environment within a single PtK2 cell, as revealed by scanning FCS (45–47). By scanning the confocal observation spot (d = 240 nm) rapidly (frequency f = 4 kHz) along a line (4 μm long) crossing the boundary of MBP-rich domains, we recorded FCS data and determined the D_{app} for each pixel (100 pixels over the line), which we then correlated with the GFP signal of the MBP-rich domains. Clearly, the lipid mobility was increased inside the domains, as indicated for SM (Fig. 4, *B* and *C*). Confirming our previous observations, the increase in mobility was higher for SM than for PE (Fig. 4 *D*) after the change from trapping diffusion of SM to fast free diffusion. Interestingly, only a small increase in mobility was observed for a fluorescent cholesterol analog (cholesterol-PEG-KK114, cholesterol labeled with the dye KK114 via a PEG linker). The partitioning of this cholesterol analog into membrane environments of different molecular order was previously determined to be neutral (i.e., with no preference for ordered versus disordered environments) and a diffusion analysis indicated free Brownian motion in PtK2 cells (47). Therefore, this probe most likely reports the average viscosity of the local membrane, which shows only a slight increase in mobility (i.e., decrease in viscosity) in the MBP domains. The stronger difference in diffusion of the SM and PE lipid

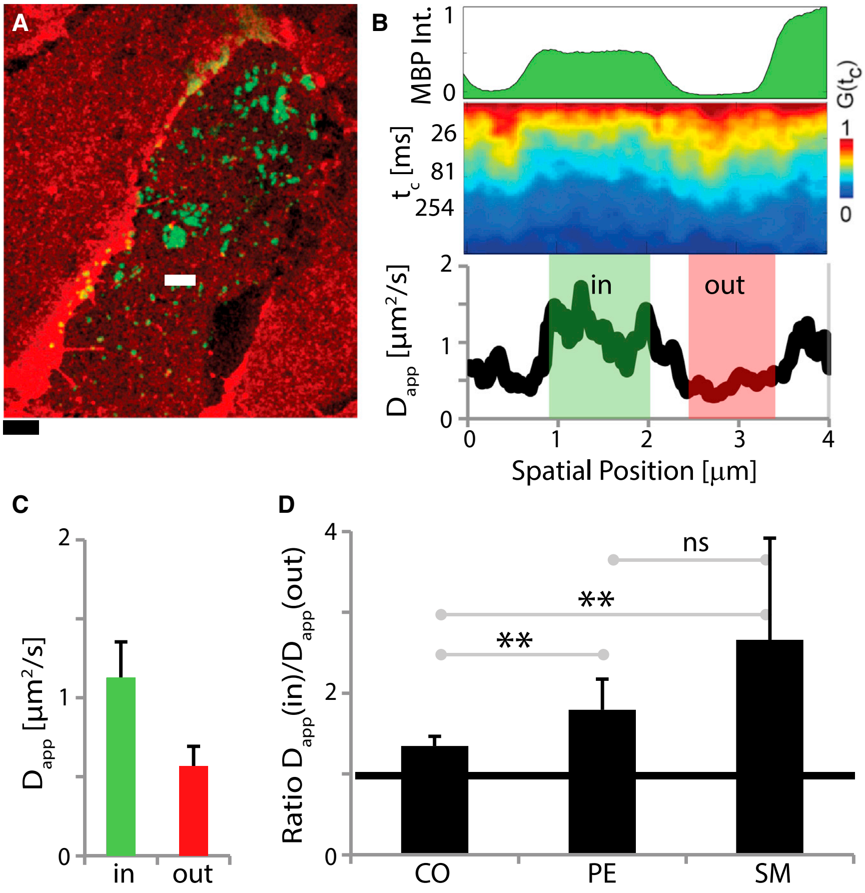

FIGURE 4 Scanning FCS directly reveals increased lipid mobility in MBP-enriched areas in a single cell. (*A*) Two-color confocal image of the basal membrane of PtK2 cells expressing GFP-TM-MBP (*green*) and incorporating the SM lipid analog (*red*). FCS data were recorded along a line crossing one or more MBP-rich domains (*white line*, length $l = 4$ μm, 60 s, scanning frequency $f = 4$ kHz). Scale bar, 4 μm. (*B*) Representative scanning-FCS data for SM: normalized intensity of GFP-MBP (*top*, *green*), correlation carpet (*middle*, correlation data $G(t_c)$ for each pixel of the scan decaying with correlation time t_c as indicated by the *red to blue* transition), and D_{app} (*bottom*), indicating increased mobility of SM within the domains (in, *green-shaded area*; enhanced GFP signal and faster decays of the correlation data) as compared with outside of the domains (out, *red-shaded area*). (*C*) Average value and standard deviation (*error bars*) of D_{app} of SM inside (in, *green*) and outside (out, *red*) of MBP-rich domains, as determined from 10 independent line scans at different positions. (*D*) Average and standard deviation (*error bars*, 10 independent measurements) of the ratio D_{app}(in)/D_{app}(out) of lipid mobility inside and outside of the MBP-rich domains for three fluorescent analogs: cholesterol (CO, cholesterol-PEG-KK114), PE, and SM. The increase in mobility when entering the MBP-rich domains was highest for SM, slightly lower for PE, and lowest for the cholesterol analog. All measurements were performed at 37°C. Statistical *p*-test: **significant; ns, nonsignificant. To see this figure in color, go online.

analogs most likely reflects the reduced protein interactions within MBP domains compared with the crowded rest of the plasma membrane.

The MBP protein may modulate lipid diffusion via distinct mechanisms, ranging from a simple exchange of the structure of the meshwork underlying the plasma membrane to, for example, reordering and sorting of the lipids that are allowed to be in the MBP domains. It was previously observed that oligodendrocytes specifically increase the lipid order (or packing) of membrane sheets by segregation of long-chain lipids into the sheets (8). Therefore, we investigated whether the presence of the MBP meshwork was also able to promote lipid packing and ordering in the domains that formed in the plasma membrane of the PtK2 cells. For this purpose, we expressed MBP fused to the red-emitting fluorescent protein mCherry in live PtK2 cells (mCherry-TM-MBP) to generate and label MBP-enriched domains. In addition, we added the polarity-sensitive membrane dye C-Laurdan, whose emission is relatively blue-shifted (~440 nm) for more ordered (or packed) membranes containing, for example, more saturated or long-chain lipids, and relatively red-shifted in more disordered membranes (~490 nm) (48). We used mCherry instead of GFP as a label of MBP to avoid cross talk with the emission of C-Laurdan and any possible energy transfer between the two fluorescent molecules. To observe changes in the emission of C-Laurdan and thus in the lipid membrane order inside and outside the MBP-enriched domains, we applied spectral imaging. This technique allowed us to investigate changes in the emission spectrum of C-Laurdan due to changes in the lipid packing for each image pixel, as highlighted previously (49).

Fig. 5, *A–E*, shows the results of the spectral imaging of the C-Laurdan emission in correlation to the signal of mCherry marking the MBP-rich domains. The emission of C-Laurdan was more red-shifted, i.e., the membrane was characterized to be more disordered in the MBP-rich domains. This is surprising, since membrane sheets in oligodendrocytes are known to have a very high membrane order (8).

One possible explanation for our result is that a large fraction of C-Laurdan is internalized into cells (50) and thus may potentially also label the internal ER membrane in the close vicinity of the plasma membrane induced by the MBP-enriched domains (Fig. 2 *A*). Therefore, we employed a second probe, SL2. Although this probe is less sensitive than C-Laurdan to lipid packing changes in terms of emission shift, it is useful in investigations of plasma membrane ordering due to its minimal cellular internalization (50). We therefore repeated the spectral imaging experiments using SL2 (Fig. 5, *F–L*). However, we observed no difference in

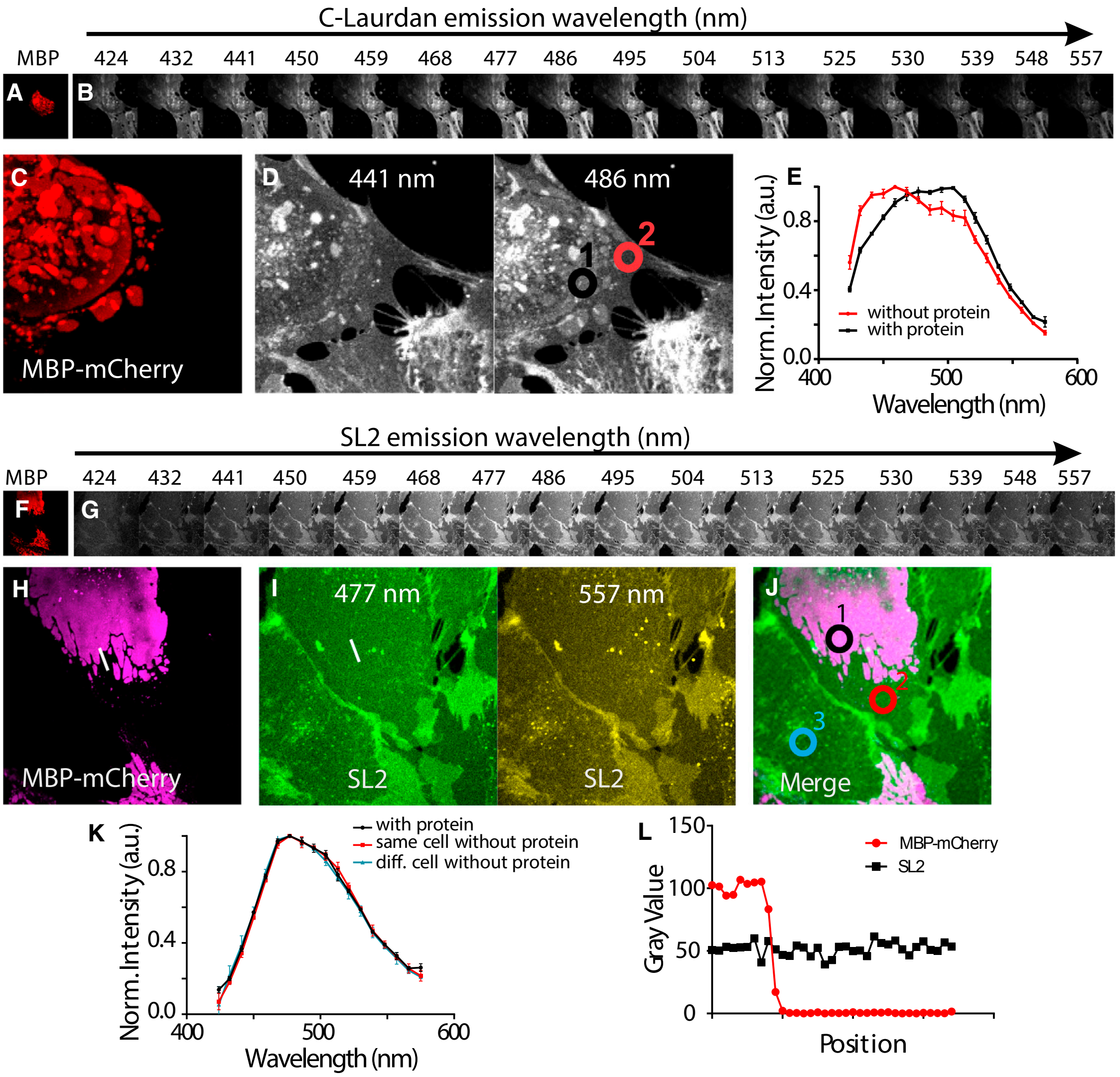

FIGURE 5 Changes in membrane order in mCherry-TM-MBP-enriched domains of PtK2 cells. (*A*) Large (40 × 40 μm^2) confocal image of the basal membrane of PtK2 cells expressing mCherry-TM-MBP, indicating the expression and positions of MBP-enriched domains (*red*). (*B*) Images of the same cells for different spectral ranges of the fluorescence emission of C-Laurdan. A slight shift of the fluorescence emission of C-Laurdan toward longer wavelengths is observed within the MBP-enriched domains. (*C* and *D*) Close-up images of (*A*) and (*B*): mCherry fluorescence (*red*, *C*) and emission of C-Laurdan at 441 nm (*D*, *left*) and 486 nm (*D*, *right*). (*E*) Fluorescence emission spectrum of C-Laurdan from areas marked by circles in (*D*): inside (circle 1, *black*) and outside (circle 2, *red*) MBP-enriched domains, revealing a shift of C-Laurdan emission toward longer wavelengths and thus a decrease in membrane order within the domains. (*F*) Large (40 × 40 μm^2) confocal image of the basal membrane of PtK2 cells expressing mCherry-TM-MBP, indicating the expression and positions of MBP-enriched domains (*red*). (*G*) Images of the same cells for different spectral ranges of the fluorescence emission of SL2. No shift of the fluorescence emission of SL2 is observed within the MBP-enriched domains. (*H–J*) Close-up of a part of the images in (*F*) and (*G*): mCherry fluorescence (*H*, *purple*), emission of SL2 at 477 nm (*I*, *left*), and 557 nm (*I*, *right*), and a merged image (*J*) of mCherry emission (*purple*) and SL2 emission at 477 nm (*gray*). (*K*) Fluorescence emission spectrum of SL2 from areas marked by circles in (*J*): inside (circle 1, *black*) and outside (circle 2, *red*) MBP-enriched domains of the same cell, and on a cell expressing no mCherry-MBP (circle 3, *blue*), revealing no shift of SL2 emission and thus no change in membrane order within the MBP-enriched domains. (*L*) This is further confirmed by plotting the intensity (arbitrarily scaled) of mCherry fluorescence (*red*) and SL2 fluorescence at 477 nm (*black*) along the white line marked in (*I*), crossing the border of the MBP-enriched domain. To see this figure in color, go online.

the fluorescence emissions of the plasma-membrane probe SL2 between MBP-rich and -poor regions (Fig. 5 *K*), indicating that MBP did not induce a major change in molecular order. In addition to the spectral shift, the absolute emission intensity of SL2 is also modulated by molecular lipid ordering (51); that is, SL2 is brighter in ordered membrane regions than in disordered membrane regions. When we tested the intensity profile in MBP-rich and -poor regions, we observed no difference (Fig. 5 *L*). Thus, in contrast to membrane sheets in oligodendrocytes, the MBP-induced domains in epithelial PtK2 cells demonstrate no changes in lipid order, indicating that segregation

of long-chain lipids into myelin sheets is a process specific to oligodendrocytes.

DISCUSSION

Previous studies have shown that the fluorescent lipid analogs SM and GalCer undergo trapping diffusion in epithelial cells and fibroblasts (29,31,33). Here, we investigated the diffusion of different fluorescent lipid analogs (PE, SM, and GalCer) in oligodendrocytes and found that diffusion in myelin sheets was substantially different from that observed in epithelial cells and fibroblasts; specifically, the mobility was increased and transient arrests were reduced. This strongly suggests that the myelin membrane composition and organization may play an important role in molecular diffusion at the cell surface. We specifically addressed the role of MBP in the dynamic organization of lipids by introducing MBP into PtK2 cells, where MBP forms micrometer-size patches between the ER and the plasma membrane. In these domains, the actin cytoskeleton is effectively replaced by a meshwork of MBP where lipid diffusion is significantly less hindered. Thus, we conclude that MBP is a key modulator of lipid diffusion in myelin.

MBP influences key aspects of the plasma membrane. It depletes proteins from the membrane by forming a diffusion barrier for proteins with large cytoplasmic domains (4,52) and replaces the cortical network of actin filaments at the cytoplasmic leaflet of the myelin membrane bilayer. In intact cells, transient molecular interactions with slow-moving or immobilized membrane constituents such as proteins have been shown to cause hindered diffusion of lipids, assisted by the cortical actin cytoskeleton (21,28,32,40). These sources of hindered diffusion are missing in MBP-enriched membrane regions and most probably explain the differences in diffusion between these membranes and the plasma membrane of fibroblasts and epithelial cells. Since MBP itself forms a network that underlies the plasma membrane, it may also restrain molecular mobility in the membrane to some extent, for example, by friction or steric effects. Nevertheless, from our findings, we conclude that the extrusion of the actin cytoskeleton and associated membrane proteins seems to be more significant in determining molecular diffusion in the plasma membrane than the interaction between the two-dimensional gel of MBP and the inner leaflet of the plasma membrane.

Another factor that could work against the tendency of faster and less-hindered diffusion in MBP-enriched membrane regions is the formation of a more densely packed and ordered lipid environment. A high degree of lipid packing and order has been observed in membrane sheets of oligodendrocytes (8). However, our experiments with spectral imaging showed that MBP expression and local enrichment did not promote reordering of lipids in the plasma membrane of PtK2 cells. This indicates that segregation of saturated long-chain lipids into membrane sheets is a process specific to oligodendrocytes, possibly requiring the specific lipid composition of myelin-forming cells. On the other hand, the difference in lipid ordering between the myelin sheets of oligodendrocytes and PtK2 MBP+ regions might explain why lipid diffusion is, in general, faster in PtK2 MBP+ than in myelin sheets.

Differences in lipid compositions may also explain why the lipid analogs diffuse much more slowly in membrane sheets of oligodendrocytes as compared with the MBP-enriched domains of the PtK2 cells. The oligodendrocyte-specific enrichment in long-chain, saturated lipids ($>C_{24}$) (6,8) increases not only the membrane order or packing but also the probability of inner-leaflet coupling, which in turn results in increased friction, viscosity, and lower lipid mobility (53). It would be interesting to study the diffusion of fluorescent lipid analogs with long-chain lipids ($>C_{24}$), but unfortunately, so far we have only been able to efficiently incorporate fluorescent lipid analogs with relatively short-chain fatty acids ($<C_{20}$) into cellular membranes.

Taking into account all of our previous STED-FCS experiments, we can conclude that the MBP-induced domains of PtK2 cells constitute the most fluid cellular-based membrane characterized so far, with free lipid diffusion comparable only to that of model membrane bilayers, but still with a 3-fold lower overall mobility (24,39). A potential cause of the still slower diffusion in myelin membrane sheets compared with pure model membrane systems may be weak interactions or molecular crowding due to residual proteins or residual actin patches (30).

Here, we did not consider the influences of membrane curvature. This may be an important issue when considering the multilamellar membrane organization of myelin surrounding an axon. However, it is important to note that the curvature of the different myelin layers varies, which may affect how the lipids diffuse. In this study, we wanted to focus on the influence of the MBP proteins on lipid membrane diffusion.

Overall, this work indicates that MBP has major impact on the organization of the plasma membrane by modulating membrane heterogeneities, such as in the supporting actin-cytoskeleton, thereby controlling membrane fluidity and heterogeneity. Clearly, additional mechanisms such as the specific lipid composition and the lamellar membrane structure must be at work in native myelin sheaths.

SUPPORTING MATERIAL

Supporting Materials and Methods and two figures are available at http://www.biophysj.org/biophysj/supplemental/S0006-3495(16)30273-9.

AUTHOR CONTRIBUTIONS

O.S. and D.M.A. designed and conducted experiments, and analyzed the data. V.M. helped with the experiments. A.H. performed the scanning FCS experiments and assisted STED-FCS recordings, E.S. performed the membrane order experiments, and F.S. and E.S. performed the experiments

on Lactadherin and GUVs. C.E., M.S., S.W.H., and A.H. designed the research. O.S., D.M.A., C.E., and M.S. wrote the manuscript. All authors contributed in discussing the data, experiments, and manuscript.

ACKNOWLEDGMENTS

We thank Tanja Gilat and Ellen Rothermel (MPI-BPC Göttingen) for helpful assistance and Shweta Aggarwal (MPI-EM Göttingen) for cloning of the GFP-TM-Lactadherin C2 domain and the GFP-TM-MBP construct.

This work was supported by grants from the German Research Foundation (DFG) (SI 746/9-1; TRR43 to M.S. and SFB755/B12 to C.E.), the E-Rare Research Program, and the Tschira-Stiftung; grants awarded to M.S. This work was also supported by a Gottfried-Wilhelm-Leibniz award from the DFG to S.W.H. E.S. is supported by EMBO long-term and Marie Skłodowska-Curie Intra-European postdoctoral fellowships. C.E., F.S., and E.S. received support from the Wellcome Trust (grant 104924/14/Z/14), the Medical Research Council (grant number MC_UU_12010/unit programs G0902418 and MC_UU_12025), Biotechnology and Biological Sciences Research Council and Engineering and Physical Sciences Research Council (grant number MR/K01577X/1), the DFG research unit 1905 "Structure and function of the peroxisomal translocon," and institutional funding from the University of Oxford.

SUPPORTING CITATIONS

References (54–57) appear in the Supporting Material.

REFERENCES

1. Sherman, D. L., and P. J. Brophy. 2005. Mechanisms of axon ensheathment and myelin growth. *Nat. Rev. Neurosci.* 6:683–690.
2. Snaidero, N., W. Möbius, …, M. Simons. 2014. Myelin membrane wrapping of CNS axons by PI(3,4,5)P3-dependent polarized growth at the inner tongue. *Cell.* 156:277–290.
3. Chrast, R., G. Saher, …, M. H. Verheijen. 2011. Lipid metabolism in myelinating glial cells: lessons from human inherited disorders and mouse models. *J. Lipid Res.* 52:419–434.
4. Aggarwal, S., L. Yurlova, …, M. Simons. 2011. A size barrier limits protein diffusion at the cell surface to generate lipid-rich myelin-membrane sheets. *Dev. Cell.* 21:445–456.
5. O'Brien, J. S. 1965. Stability of the myelin membrane. *Science.* 147:1099–1107.
6. O'Brien, J. S., and E. L. Sampson. 1965. Lipid composition of the normal human brain: gray matter, white matter, and myelin. *J. Lipid Res.* 6:537–544.
7. O'Brien, J. S., and E. L. Sampson. 1965. Fatty acid and fatty aldehyde composition of the major brain lipids in normal human gray matter, white matter, and myelin. *J. Lipid Res.* 6:545–551.
8. Yurlova, L., N. Kahya, …, M. Simons. 2011. Self-segregation of myelin membrane lipids in model membranes. *Biophys. J.* 101:2713–2720.
9. Hartline, D. K., and D. R. Colman. 2007. Rapid conduction and the evolution of giant axons and myelinated fibers. *Curr. Biol.* 17:R29–R35.
10. Harauz, G., N. Ishiyama, …, C. Farès. 2004. Myelin basic protein-diverse conformational states of an intrinsically unstructured protein and its roles in myelin assembly and multiple sclerosis. *Micron.* 35:503–542.
11. Boggs, J. M. 2006. Myelin basic protein: a multifunctional protein. *Cell. Mol. Life Sci.* 63:1945–1961.
12. Kattnig, D. R., T. Bund, …, D. Hinderberger. 2012. Lateral self-assembly of 18.5-kDa myelin basic protein (MBP) charge component-C1 on membranes. *Biochim. Biophys. Acta.* 1818:2636–2647.
13. Aggarwal, S., N. Snaidero, …, M. Simons. 2013. Myelin membrane assembly is driven by a phase transition of myelin basic proteins into a cohesive protein meshwork. *PLoS Biol.* 11:e1001577.
14. Lee, D. W., X. Banquy, …, J. N. Israelachvili. 2014. Lipid domains control myelin basic protein adsorption and membrane interactions between model myelin lipid bilayers. *Proc. Natl. Acad. Sci. USA.* 111:E768–E775.
15. Simons, M., N. Snaidero, and S. Aggarwal. 2012. Cell polarity in myelinating glia: from membrane flow to diffusion barriers. *Biochim. Biophys. Acta.* 1821:1146–1153.
16. Nawaz, S., P. Sánchez, …, M. Simons. 2015. Actin filament turnover drives leading edge growth during myelin sheath formation in the central nervous system. *Dev. Cell.* 34:139–151.
17. Zuchero, J. B., M. M. Fu, …, B. A. Barres. 2015. CNS myelin wrapping is driven by actin disassembly. *Dev. Cell.* 34:152–167.
18. Kirschner, D. A., H. Inouye, and R. A. Saavedra. 1996. Membrane adhesion in peripheral myelin: good and bad wraps with protein P0. *Structure.* 4:1239–1244.
19. Maggio, B., G. A. Borioli, …, N. Wilke. 2008. Composition-driven surface domain structuring mediated by sphingolipids and membrane-active proteins. Above the nano- but under the micro-scale: mesoscopic biochemical/structural cross-talk in biomembranes. *Cell Biochem. Biophys.* 50:79–109.
20. Singer, S. J., and G. L. Nicolson. 1972. The fluid mosaic model of the structure of cell membranes. *Science.* 175:720–731.
21. Owen, D. M., D. Williamson, …, K. Gaus. 2009. Quantitative microscopy: protein dynamics and membrane organisation. *Traffic.* 10:962–971.
22. He, H. T., and D. Marguet. 2011. Detecting nanodomains in living cell membrane by fluorescence correlation spectroscopy. *Annu. Rev. Phys. Chem.* 62:417–436.
23. Eggeling, C. 2015. Super-resolution optical microscopy of lipid plasma membrane dynamics. *Essays Biochem.* 57:69–80.
24. Kusumi, A., C. Nakada, …, T. Fujiwara. 2005. Paradigm shift of the plasma membrane concept from the two-dimensional continuum fluid to the partitioned fluid: high-speed single-molecule tracking of membrane molecules. *Annu. Rev. Biophys. Biomol. Struct.* 34:351–378.
25. Douglass, A. D., and R. D. Vale. 2005. Single-molecule microscopy reveals plasma membrane microdomains created by protein-protein networks that exclude or trap signaling molecules in T cells. *Cell.* 121:937–950.
26. van Meer, G., and W. L. Vaz. 2005. Membrane curvature sorts lipids. Stabilized lipid rafts in membrane transport. *EMBO Rep.* 6:418–419.
27. Lingwood, D., H. J. Kaiser, …, K. Simons. 2009. Lipid rafts as functional heterogeneity in cell membranes. *Biochem. Soc. Trans.* 37:955–960.
28. Head, B. P., H. H. Patel, and P. A. Insel. 2014. Interaction of membrane/lipid rafts with the cytoskeleton: impact on signaling and function: membrane/lipid rafts, mediators of cytoskeletal arrangement and cell signaling. *Biochim. Biophys. Acta.* 1838:532–545.
29. Eggeling, C., C. Ringemann, …, S. W. Hell. 2009. Direct observation of the nanoscale dynamics of membrane lipids in a living cell. *Nature.* 457:1159–1162.
30. Kusumi, A., Y. M. Shirai, …, T. K. Fujiwara. 2010. Hierarchical organization of the plasma membrane: investigations by single-molecule tracking vs. fluorescence correlation spectroscopy. *FEBS Lett.* 584:1814–1823.
31. Mueller, V., C. Ringemann, …, C. Eggeling. 2011. STED nanoscopy reveals molecular details of cholesterol- and cytoskeleton-modulated lipid interactions in living cells. *Biophys. J.* 101:1651–1660.
32. Mueller, V., A. Honigmann, …, C. Eggeling. 2013. FCS in STED microscopy: studying the nanoscale of lipid membrane dynamics. *Methods Enzymol.* 519:1–38.
33. Sahl, S. J., M. Leutenegger, …, C. Eggeling. 2010. Fast molecular tracking maps nanoscale dynamics of plasma membrane lipids. *Proc. Natl. Acad. Sci. USA.* 107:6829–6834.

34. Kusumi, A., T. K. Fujiwara, …, K. G. Suzuki. 2012. Dynamic organizing principles of the plasma membrane that regulate signal transduction: commemorating the fortieth anniversary of Singer and Nicolson's fluid-mosaic model. *Annu. Rev. Cell Dev. Biol.* 28:215–250.

35. Andrade, D. M., M. P. Clausen, …, C. Eggeling. 2015. Cortical actin networks induce spatio-temporal confinement of phospholipids in the plasma membrane—a minimally invasive investigation by STED-FCS. *Sci. Rep.* 5:11454.

36. Goñi, F. M., L. R. Montes, and A. Alonso. 2012. Phospholipases C and sphingomyelinases: lipids as substrates and modulators of enzyme activity. *Prog. Lipid Res.* 51:238–266.

37. Hell, S. W., and J. Wichmann. 1994. Breaking the diffraction resolution limit by stimulated emission: stimulated-emission-depletion fluorescence microscopy. *Opt. Lett.* 19:780–782.

38. Kastrup, L., H. Blom, …, S. W. Hell. 2005. Fluorescence fluctuation spectroscopy in subdiffraction focal volumes. *Phys. Rev. Lett.* 94: 178104.

39. Honigmann, A., V. Mueller, …, C. Eggeling. 2013. STED microscopy detects and quantifies liquid phase separation in lipid membranes using a new far-red emitting fluorescent phosphoglycerolipid analogue. *Faraday Discuss.* 161:77–89.

40. Honigmann, A., S. Sadeghi, …, R. Vink. 2014. A lipid bound actin meshwork organizes liquid phase separation in model membranes. *eLife.* 3:e01671.

41. Sezgin, E., I. Levental, …, P. Schwille. 2012. Partitioning, diffusion, and ligand binding of raft lipid analogs in model and cellular plasma membranes. *Biochim. Biophys. Acta.* 1818:1777–1784.

42. Wawrezinieck, L., H. Rigneault, …, P. F. Lenne. 2005. Fluorescence correlation spectroscopy diffusion laws to probe the submicron cell membrane organization. *Biophys. J.* 89:4029–4042.

43. Sahl, S. J., M. Leutenegger, …, C. Eggeling. 2014. High-resolution tracking of single-molecule diffusion in membranes by confocalized and spatially differentiated fluorescence photon stream recording. *ChemPhysChem.* 15:771–783.

44. Yeung, T., G. E. Gilbert, …, S. Grinstein. 2008. Membrane phosphatidylserine regulates surface charge and protein localization. *Science.* 319:210–213.

45. Ruan, Q., M. A. Cheng, …, W. W. Mantulin. 2004. Spatial-temporal studies of membrane dynamics: scanning fluorescence correlation spectroscopy (SFCS). *Biophys. J.* 87:1260–1267.

46. Ries, J., and P. Schwille. 2006. Studying slow membrane dynamics with continuous wave scanning fluorescence correlation spectroscopy. *Biophys. J.* 91:1915–1924.

47. Honigmann, A., V. Mueller, …, C. Eggeling. 2014. Scanning STED-FCS reveals spatiotemporal heterogeneity of lipid interaction in the plasma membrane of living cells. *Nat. Commun.* 5:5412.

48. Kim, H. M., H. J. Choo, …, B. R. Cho. 2007. A two-photon fluorescent probe for lipid raft imaging: C-laurdan. *ChemBioChem.* 8:553–559.

49. Sezgin, E., D. Waithe, …, C. Eggeling. 2015. Spectral imaging to measure heterogeneity in membrane lipid packing. *ChemPhysChem.* 16:1387–1394.

50. Sezgin, E., T. Sadowski, and K. Simons. 2014. Measuring lipid packing of model and cellular membranes with environment sensitive probes. *Langmuir.* 30:8160–8166.

51. Lim, C. S., H. J. Kim, …, B. R. Cho. 2011. A two-photon turn-on probe for lipid rafts with minimum internalization. *ChemBioChem.* 12:392–395.

52. Pedraza, L., J. K. Huang, and D. R. Colman. 2001. Organizing principles of the axoglial apparatus. *Neuron.* 30:335–344.

53. Rashid, R., S. M. Chee, …, T. Wohland. 2015. Macromolecular crowding gives rise to microviscosity, anomalous diffusion and accelerated actin polymerization. *Phys. Biol.* 12:034001.

54. Fitzner, D., A. Schneider, …, M. Simons. 2006. Myelin basic protein-dependent plasma membrane reorganization in the formation of myelin. *EMBO J.* 25:5037–5048.

55. Martin, O. C., and R. E. Pagano. 1994. Internalization and sorting of a fluorescent analogue of glucosylceramide to the Golgi apparatus of human skin fibroblasts: utilization of endocytic and nonendocytic transport mechanisms. *J. Cell Biol.* 125:769–781.

56. Clausen, M. P., S. Galiani, …, C. Eggeling. 2013. Pathways to optical STED microscopy. *NanoBioImaging.* 1:1–12.

57. Chiantia, S., N. Kahya, …, P. Schwille. 2006. Effects of ceramide on liquid-ordered domains investigated by simultaneous AFM and FCS. *Biophys. J.* 90:4500–4508.

Biophysical Journal, Volume *110*

Supplemental Information

Reorganization of Lipid Diffusion by Myelin Basic Protein as Revealed by STED Nanoscopy

Olena Steshenko, Débora M. Andrade, Alf Honigmann, Veronika Mueller, Falk Schneider, Erdinc Sezgin, Stefan W. Hell, Mikael Simons, and Christian Eggeling

Supporting Material

MATERIALS AND METHODS

Cell culture

Primary oligodendrocyte culture

Primary cultures of oligodendrocytes were prepared from postnatal day 1 mouse brains as described previously (1). In brief, cellular mixture from trypsinized mouse brains was grown in poly-L-lysine (PLL) coated flasks in Basal Medium Eagle (BME) medium supplemented with 10% horse serum and 100 U/mL each of penicillin and streptomycin. After 7 – 10 days, oligodendroglial progenitors growing on top of a layer of astrocytes were shaken off and cultured further in SuperSato medium on PLL-coated dishes or round coverslips (d = 18 mm) (R.Langenbrinck, Labor- und Medizintechnik, Emmendingen, Germany).

PtK2 epithelial cell line

Cells were grown in flasks with 10 ml of the PtK2-Sato medium. To prevent overgrowth, cells were split once in 6 – 7 days onto a fresh flask. For the imaging, cells were split onto 18 mm coverslips (non-treated with acid and not-covered with PLL), in 1 ml of PtK2-Sato.

PtK2-MBP assay

An assay of the reconstitution of the MBP zipping properties within the epithelial PtK2 cell line was performed as described (2). In brief, the MBP-GFP-TM or MBP-mCherry-TM chimeric construct, containing MBP, transmembrane domain, GFP or mCherry and ER-retention signal, was designed and cloned. Subsequently, PtK2 cells were transfected with this construct and subjected to the further analysis after 18 h post-transfection. Cells, positive to the transfection, were recognised due to the GFP-signal.

PtK2 Lactadherin assay

Transfection, labelling and data acquisition were performed as for cells transfected with MBP-GFP. PtK2 cells were grown on a 25 mm cover slips to a confluency of about 70 %. GFP-TM-Lactadherin C2 domain was transfected using Lipofectamine 3000 (Life Technologies) according to the manufacturer’s protocol. After washing with L15 media, cells were labelled with Atto647N-SM in L15 at a total concentration of 400 µg/mL for 15 minutes at room temperature. After washing with L15 for 3 times, STED-FCS data were acquired in a time window of 1 hour.

Immunolabelling

Primary antibodies used in the study are: rabbit anti-MBP (1:300) (DakoCytomat, Carpinteria, CA), Phalloidin-Rhodamine (F-actin detection) (1:200) (Invitrogen, Munich, Germany), WGA-488 (for lectin detection) (1:200) from (Invitrogen, Munich, Germany). Secondary

fluorophore-conjugated antibodies (Dianova, Hamburg, Germany) were used in 1:200 dilution.

BSA-coupling of fluorescent lipid analogues

To enable incorporation into cellular membranes, lipids analogues were primarily coupled to bovine serum albumin (BSA) as described in (3) with slight modifications. 75 nmol of lipid analogues were first reconstituted in 1:1 chloroform/methanol solution; liquid was further aspirated and lipid films were redissolved in 10 µl of absolute ethanol and vortexed vigorously. Defatted BSA (in Dulbecco's Modified Eagle Medium DMEM without phenol-red, buffered with 10 mM HEPES) was added in equimolar concentration to the lipids. Solution was centrifuged at the maximum speed for 3 min to remove possible aggregates. Supernatant was stored at -20C°.

Incorporation of the lipid analogues

To incorporate lipids into cellular membranes, cells were first washed with 10 mM HEPES-buffered DMEM medium without phenol-red (HDMEM, ice-cold). Next, BSA-lipid complexes, diluted in HDMEM, were added in the appropriate concentrations onto cells in a wet-chamber on ice for 30 min. After incubation, cells were briefly washed with HDMEM and immediately imaged. The fluorescent lipophilic organic dye Atto647N (excitation max at 645 nm, emission max at 670 nm; Atto-Tec, Siegen, Germany) was used as a marker of the incorporated lipids (4-7). Lipids used for the diffusion experiments were as following (5): **PE** – *N*-(Atto647N)-1,2-dihexadecanoyl-*sn*-glycero-3-phosphoethanolamine; **SM** – *N*-(Atto647N)-sphingomyelin; **GalCer –** *N*-(Atto647N)-galactosylsphingosine (Psychosine). Lipids were labelled either at the head group (PE), meaning at the water phase or lipid-water interphase, or via replacement of one the native lipid acyl chains by the short acyl chain carrying a dye (acyl-chain replacement **–** SM, GalC). Throughout the text these lipids are referred simply as PE, SM, GalCer.

Preparation of giant-unilamellar-vesicles (GUVs)

GUVs were prepared by electroformation as described in (Sezgin et al BBA or Garcia-Saez 2009). Briefly, 1 mg/mL DOPC (Avanti Polar Lipids) was spread on platinum wire, evaporated and dipped into 300 mM sucrose solution. GUVs formed during exposure to an electric field of 10 Hz (2V). Lipid analogs, namely Atto647N-DPPE, Atto647N-GalCer and Atto647N-SM were added after formation to a final concentration of about 20, 30 and 100 µg/mL, respectively. GUVs were dropped onto a BSA coated cover slip. The diffusion measurements were performed on the bottom membrane of the vesicles using 640 nm excitation and 650 long pass emission. At least ten different GUVs were measured.

STED-FCS experimental setup

All experiments were performed on a confocal custom-built STED microscope (7) or a STED-modified Abberior Instrument’s Resolft microscope (Abberior Instruments GmbH, Göttingen, Germany) (8). On the first microscope, the confocal unit of the STED nanoscope consisted of an excitation and detection beam path. A fiber-coupled pulsed laser diode

operating at λ_{exc} = 635 nm with a pulse length of 80 ps (LDH-P-635, PicoQuant, Berlin, Germany) was used for excitation of the red fluorescence. After leaving the fiber, the excitation beam was expanded and focused into the sample using an oil immersion objective (HCXPLAPO 100x, NA = 1.4, Leica Microsystems). The fluorescence emitted by the sample was collected by the same objective lens and separated from the excitation light by a custom-designed dichroic mirror (AHF Analysentechnik, Tuebingen, Germany). In the following, the fluorescence was focused onto a multi-mode fiber splitter (Fiber Optic Network Technology, Surrey, Canada). The aperture of the fiber acted as a confocal pinhole of 0.78 of the diameter of the back-projected Airy disk. In addition, the fiber 50:50 split the fluorescence signal, which was then detected by two single-photon counting modules (APD, SPCM-AQR-13-FC, Perkin Elmer Optoelectronics, Fremont, CA). The detector signals were acquired by a single-photon-counting PC card (SPC 830, Becker&Hickl, Berlin, Germany). The confocal setup was extended by integrating a STED laser beam. A modelocked Titanium:Sapphire laser (Ti:Sa, MaiTai, Spectra-Physics, Mountain View, USA) acted as the STED laser emitting sub-picosecond pulses around λ_{STED} = 780 nm with a repetition rate of 80 MHz. The pulses of the STED laser were stretched to 250-350 ps by dispersion in a SF6 glass rod of 50 cm length and a 120 m long polarization maintaining single-mode fiber (PMS, OZ Optics, Ontario, CA). After the fiber, the STED beam passed through a polymeric phase plate (RPC Photonics, Rochester, NY) which introduced a linear helical phase ramp $0 \leq \Phi \leq 2\pi$ across the beam diameter. This wavefront modification gave rise to the doughnut-shaped focal intensity distribution featuring a central intensity zero. The temporal synchronization of the excitation and STED pulses was achieved by triggering the pulses of the excitation laser using the trigger signal from an internal photodiode inside the STED laser and a home-built electronic delay unit, which allowed a manual adjustment of the delay with a temporal resolution of 25 ps. The circular polarization of the STED and excitation laser light in the focal plane was maintained by a combination of $\lambda/2$ and $\lambda/4$ retardation plates in both beam paths (B. Halle, Berlin, Germany).

Integration of a fast scanning unit enabled rapid scanning of the excitation and STED beam across the sample plane (Fig. S1B). A digital galvanometric two mirror scanning unit (Yanus digital scan head, TILL Photonics, Gräfeling, Germany) was used for this purpose. The combination of an achromatic scan lens and a tube lens in a 4f-configuration (f = 50 mm and f = 240 mm, Leica, Wetzlar, Germany) realized a stationary beam position in the back aperture of the objective, preventing peripheral darkening within the focal plane at large scan ranges, such as vignetting. The maximal frequency of the Yanus scanner depended on the scan amplitude and varied between 2 - 6 kHz for scan amplitudes up to 150 µm, respectively. The hardware and data acquisition was controlled by the software ImSpector (http://www.imspector.de/). The synchronization of the beam-scanning for scanning-FCS data acquisition has been described in detail elsewhere (7).

STED-FCS cellular measurements

Day 5 oligodendrocytes and PtK2 cells were grown on standard glass coverslips (diameter 18 mm, no. 1.5 thickness) to a confluence of about 80% at 37C° in a water-saturated atmosphere of 5% CO_2 in the air. Incorporation of the fluorescent lipid analogues (lipid-Atto647N) by cells was accomplished via BSA coupling. Measurements were performed at 37C° in HDMEM.

As detailed previously (5, 7, 8), we ensured a non-detectable (at least by STED-FCS) influence by the dye label, and that the observation times were given by the focal transit times and not shortened due to photobleaching (by measuring at low enough excitation intensities), and that biasing effects by the excitation or STED light due to photobleaching, heating or other (non-linear) effects and diffusion of non-integrated lipids (or dye tags) could

be excluded. Also, we can exclude a significant amount of immobilized lipid analogues. Such fraction would have become visible at the beginning of an FCS measurement by a decrease in the overall fluorescence intensity, which we have not detected. In addition, previous FRAP (Fluorescence Recovery After Photobleaching) experiments on SM and PE in PtK2 cells showed an immobile fraction of < 5% (5, 7, 8).

We assessed the dynamics of fluorescent lipid analogues by placing the focused co-centred excitation and STED beams on random positions at the plasma membrane and completed all measurements before any significant morphological changes in the cell could occur. All measurements were carried out during 10 s, providing a correlation time longer than two orders of magnitude times the typical transient time of the labelled lipids through the confocal observation area. The calculated apparent diffusion coefficient for each given effective beam diameter resulted from the average of measurements performed in different cells. For each cell analysed, the apparent diffusion coefficient for each given effective beam diameter resulted from the average of at least 2 and up to 8 repetitions of the STED-FCS measurement at the given cell. For each given effective beam diameter, the averaged values obtained from individual cells were then averaged so that the correspondent standard error of the mean reflects the variance among cells.

STED-FCS analysis

We fitted all correlation data $G(t_c)$ with a model for two-dimensional diffusion,

$$G(t_c) = DC + \frac{1}{N}\frac{1}{1+t_c/t_D} + Texp(-\frac{t_c}{t_T})$$

Where t_c is the correlation time, N is the average number of fluorescent particles in the observation spot, t_D is the average transit time of the fluorescent molecules through the observation spot, and T and t_T are the amplitude and characteristic correlation time accounting for the population kinetics of the fluorescent molecules' dark triplet state. Correlation data were recorded with lipid concentrations resulting in a temporal average of particle number $N \approx 0.5$–10 fluorescent particles for the highest STED depletion and $N \approx 10$–200 fluorescent particles for confocal recordings. The correspondent apparent diffusion coefficient D was calculated based on the knowledge of the observation diameter d of the observation spot, provided by the calibration of the system (see below), $D = d^2/(8 \cdot t_D \cdot ln(2))$. To obtain a final value of D for each measurement condition (different STED powers or diameters d and different lipids), values determined from measurements taken on the same cell were first averaged and then a final average calculated from averaging all average values of all (n) individual cells measured in this condition. We usually recorded at least 5 curves per cell for n = 10-20 cells. The standard error of the mean (s.e.m.) was calculated from the deviations in-between cells.

STED-FCS calibration

Supported lipid bilayers (SLBs) were used to calibrate the STED-FCS setup. The SLBs were prepared based on a procedure described elsewhere (9). Briefly, the lipid DOPC (1,2-dioleoyl-sn-glycero-3-phosphocholine, Avanti, Alabaster, AL) and a fluorescent lipid analogue (DPPE-Atto647N, Atto-Tec, Siegen, Germany) were mixed in organic solvents (Chloroform/MeOH 1:1) at a lipid concentration of 1 mg/ml. The ratio of labelled lipids per non-labelled ones was approximately 1:10000. 50 ml of such solution were dropped onto a piranha-cleaned standard microscope cover glass (diameter 22 mm, no. 1.5 thickness) and spin-coated at 60 Hz for about one minute. The cover glass was then placed in a microscopy

chamber and subsequently the dry thin lipid film was rehydrated with 500 µl buffer solution (150 mM NaCl, 10 mM HEPES). Such bilayers were stable for several hours.

Calibration of the diameter $d(P_{STED})$ of the effective focal fluorescence spots formed by a certain STED power P_{STED} was performed by STED-FCS measurements of fluorescent lipid analogues in supported lipid bilayers (SLBs), for that such lipid bilayers provide a two-dimensional free diffusing system of molecules, labelled with the same fluorophore used in our cellular experiments. The confocal observation diameters being determined by fluorescent beads ($d(P_{STED} = 0) \approx 240$nm), the other effective diameters $d(P_{STED} \neq 0)$ can be calculated by performing STED-FCS measurements on supported lipid bilayers and using the relation:

$$\frac{d(P_{STED}=0)}{d(P_{STED})} = \sqrt{\frac{t_D(P_{STED}=0)}{t_D(P_{STED})}}$$

where t_D stands for the average transient times correspondent to each given P_{STED}. The relation above stems from the fact that the lipids in SLBs undergo two-dimensional free diffusion, so that the diffusion time scales proportionally with the diffusion area.

Spectral Imaging

Spectral imaging of the different membrane samples were performed on a Zeiss LSM 780 confocal microscope equipped with a 32-channel GaAsP detector array, as highlighted previously (10). Laser light at 405 nm was selected for fluorescence excitation of C-Laurdan and SL2. The lambda detection range was set between 415 nm and 691 nm. Despite the fact that wavelength intervals of down to 4 nm can be chosen for the individual detection channels, we have set these intervals to 8.9 nm, which allowed the simultaneous coverage of the whole spectrum with the 32 detection channels. The images were saved in .lsm file format.

The spectra for each image pixel were obtained from the intensity values of the 32 different detection channels by using the ImageJ plug-in “Stacks-T functions-Intensity vs. Time Monitor”, which has been described in detail previously (10) and can be downloaded at https://github.com/dwaithe/GP-plugin. We usually applied the plugin only on pixels within a region of interest of the acquired images. Background signal was determined by applying the same plugin on a dark region (of the same size) of the image, and subtracted from the signal from the region of interest.

Confocal Microscopy

Confocal microscopic images were acquired with a Leica DMIRE2 microscope and a Leica TCS SP2 AOBS confocal laser scanning setup (Leica Microsystems, Mannheim, Germany). 40X NA 1.25 or 63X NA 1.4 oil plan-apochromat objectives (Leica Microsystems, Mannheim, Germany) were used for image acquisition.

Image Processing and Statistical Analysis

Images were processed and analysed with the public domain Java-based image processing software ImageJ (created by Rasband, W.S., National Institutes of Health, Bethesda,

Maryland, USA), or with a MBF collection of plugins for ImageJ ("MBF ImageJ for Microscopy").

Statistical analysis was performed using MS Office Excel and GraphPad Prism software; unpaired student t-test was used as a statistical test.

SUPPORTING REFERENCES

1. Fitzner D, Schneider A, Kippert A, Mobius W, Willig KI, Hell SW, Bunt G, Gaus K, Simons M. Myelin basic protein-dependent plasma membrane reorganization in the formation of myelin. EMBO J 2006;25(5037-5048):5037-5048.
2. Aggarwal S, Snaidero N, Pahler G, Frey S, Sanchez P, Zweckstetter M, Janshoff A, Schneider A, Weil MT, Schaap IA, Gorlich D, Simons M. Myelin membrane assembly is driven by a phase transition of myelin basic proteins into a cohesive protein meshwork. PLoS Biol 2013;11(6):e1001577.
3. Martin OC, Pagano RC. Internalization and sorting of a fluorescent analogue of glucosylceramide to the Golgi apparatus of human skin fibroblasts: utilization of endocytic and nonendocytic transport mechanisms. J Cell Biol 1994;125:769-781.
4. Eggeling C, Ringemann C, Medda R, Schwarzmann G, Sandhoff K, Polyakova S, Belov VN, Hein B, von Middendorff C, Schonle A, Hell SW. Direct observation of the nanoscale dynamics of membrane lipids in a living cell. Nature 2009;457:1159-1162.
5. Mueller V, Ringemann C, Honigmann A, Schwarzmann G, Medda R, Leutenegger M, Polyakova S, Belov VN, Hell SW, Eggeling C. STED nanoscopy reveals molecular details of cholesterol- and cytoskeleton-modulated lipid interactions in living cells. Biophys J 2011;101:1651-1660.
6. Mueller V, Honigmann A, Ringemann C, Medda R, Schwarzmann G, Eggeling C. FCS in STED Microscopy: Studying the Nanoscale of Lipid Membrane Dynamics. In: Tetin SY, editor. Methods Enzymol. Burlington: Academic Press: Elsevier; 2013. p. 1-38.
7. Honigmann A, Mueller V, Ta H, Schoenle A, Sezgin E, Hell SW, Eggeling C. Scanning STED-FCS reveals spatio-temporal heterogeneity of lipid interaction in the plasma membrane of living cells. Nature Communications 2014;5:5412.
8. Clausen MP, Galiani S, Bernardino de la Serna J, Fritzsche M, Chojnacki J, Gehmlich K, Lagerholm BC, Eggeling C. Pathways to optical STED microscopy. NanoBioImaging 2013;1(1):1-12.
9. Chiantia S, Kahya N, Ries J, Schwille P. Effects of Ceramide on Liquid-Ordered Domains Investigated by Simultaneous AFM and FCS. Biophys J 2006;90:4500–4508.
10. Sezgin E, Waithe D, Bernardino de la Serna J, Eggeling C. Spectral Imaging to Measure Heterogeneity in Membrane Lipid Packing. Chemphyschem 2015;16(7):1387-1394.

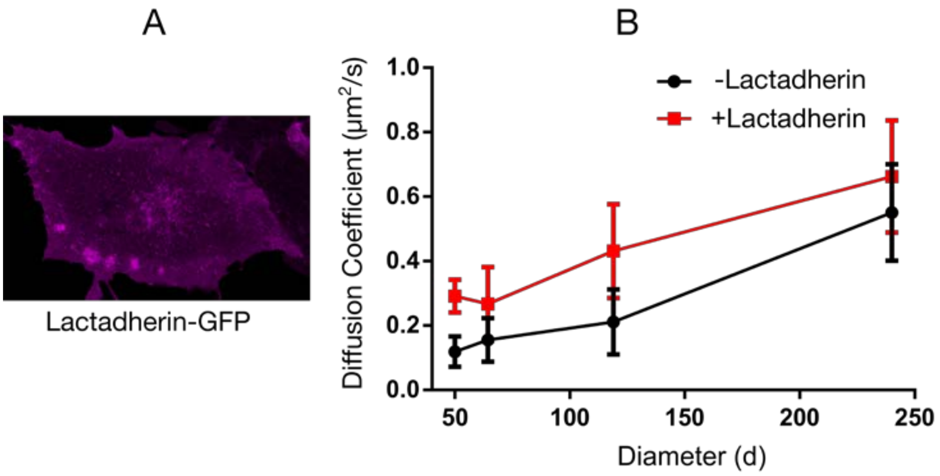

Supplementary Figure 1: Diffusion of SM with and without Lactadherin C2. (**A**) Exemplary image of the bottom membrane of GFP-TM-Lactadherin C2 domain transfected Ptk2 cells (purple: GFP signal). (**B**) STED-FCS data of SM with (+) and without (-) Lactadherin C2, indicating slightly faster diffusion in the case of GFP-TM-Lactadherin expression, but more importantly trapping in both cases. Error bars correspond to the standard deviation determined from two independent data sets including several cells.

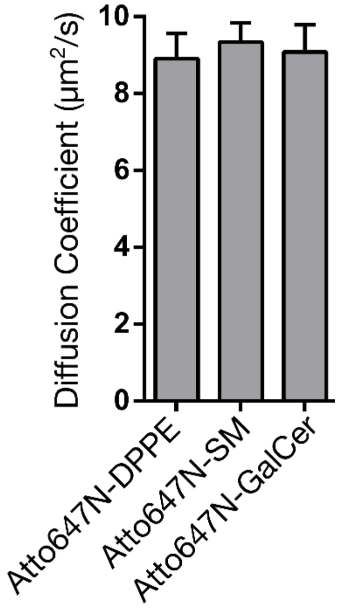

Supplementary Figure 2: Diffusion coefficient determined from confocal FCS measurements of Atto647N-labeled PE, SM and GalCer in GUVs, indicating statistically similar diffusion coefficients. Error bars represent standard deviation determined from 10 independent measurements.
